# The Transcription Factor STAT-1 Couples Macrophage Synthesis of 25-Hydroxycholesterol to the Interferon Antiviral Response

**DOI:** 10.1016/j.immuni.2012.11.004

**Published:** 2013-01-24

**Authors:** Mathieu Blanc, Wei Yuan Hsieh, Kevin A. Robertson, Kai A. Kropp, Thorsten Forster, Guanghou Shui, Paul Lacaze, Steven Watterson, Samantha J. Griffiths, Nathanael J. Spann, Anna Meljon, Simon Talbot, Kathiresan Krishnan, Douglas F. Covey, Markus R. Wenk, Marie Craigon, Zsolts Ruzsics, Jürgen Haas, Ana Angulo, William J. Griffiths, Christopher K. Glass, Yuqin Wang, Peter Ghazal

**Affiliations:** 1 Division of Pathway Medicine and Edinburgh Infectious Diseases, University of Edinburgh, Edinburgh EH16 4SB, UK; 2 SynthSys (Synthetic and Systems Biology), University of Edinburgh, The King’s Buildings, Edinburgh EH9 3JD, UK; 3 Departments of Biochemistry and Biological Sciences, Yong Loo Lin School of Medicine, National University of Singapore, Singapore 117597; 4 Department of Molecular Biology, University of California, San Diego, La Jolla, CA 92093, USA; 5 Institute of Mass Spectrometry, College of Medicine, Grove Building, Swansea University, Singleton Park, Swansea SA2 8PP, UK; 6 Department of Developmental Biology, Washington University School of Medicine, St. Louis, MO 63011, USA; 7 Max von Pettenkofer-Institut, Ludwig-Maximilians-Universität München, Genzentrum, Feodor Lynen Str. 25, 81377 Munich, Germany; 8 Facultad de Medicina, Institut d’Investigacions Biomèdiques August Pi i Sunyer, Rosselló 149-153, Barcelona 08036, Spain

## Abstract

Recent studies suggest that the sterol metabolic network participates in the interferon (IFN) antiviral response. However, the molecular mechanisms linking IFN with the sterol network and the identity of sterol mediators remain unknown. Here we report a cellular antiviral role for macrophage production of 25-hydroxycholesterol (cholest-5-en-3β,25-diol, 25HC) as a component of the sterol metabolic network linked to the IFN response via Stat1. By utilizing quantitative metabolome profiling of all naturally occurring oxysterols upon infection or IFN-stimulation, we reveal 25HC as the only macrophage-synthesized and -secreted oxysterol. We show that 25HC can act at multiple levels as a potent paracrine inhibitor of viral infection for a broad range of viruses. We also demonstrate, using transcriptional regulatory-network analyses, genetic interventions and chromatin immunoprecipitation experiments that Stat1 directly coupled *Ch25h* regulation to IFN in macrophages. Our studies describe a physiological role for 25HC as a sterol-lipid effector of an innate immune pathway.

## Graphical abstract

**Graphical Abstract:**

**Figure:**
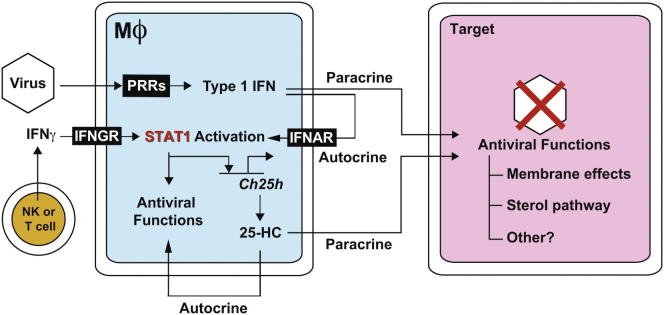

**Highlights:**

► Macrophage PRR sensing of virus or IFN activation induce 25HC synthesis and secretion ► Stat1 rapidly binds and activates the promoter of cholesterol-25-hydroxylase (Ch25h) ► 25HC exerts multilevel antiviral function for a range of different viruses ► 25HC is an intrinsic paracrine and autocrine effector of the IFN antiviral response

## Introduction

Metabolism and innate immunity are increasingly viewed as being connected and pathway mechanisms exist for sharing resources and cross-regulation (Im et al., 2011).

Cholesterol is a vital component of cellular membranes and is critical to a broad range of cellular functions. As a result, elaborate homeostatic mechanisms have evolved to control sterol metabolism at multiple levels within and outside the cell (Horton et al., 2002). Importantly, several of these mechanisms involve 25-hydroxycholesterol (25HC). The synthesis of transcripts encoding virtually all enzymes of the sterol biosynthesis pathway is regulated by the transcription factor Srebp2. Moreover, the Srebp2 processing pathway, responsible for endoplasmic reticulum retention of the protein or proteolytic activation and translocation to the nucleus, is subject to inhibition by 25HC (Gong et al., 2006; Radhakrishnan et al., 2007; Yabe et al., 2002). In the mevalonate arm of the pathway, HMGCR serves as a key control point (Rodwell et al., 1976) and is further regulated by oxysterols (in particular 25HC) through proteasomal degradation elicited by INSIG1. In addition, 25HC may also regulate cholesterol homeostasis through its ability to activate liver X receptors (LXR) and a variety of other isoprenoid and sterol metabolic intermediates can negatively feedback on sterol biosynthesis (Espenshade and Hughes, 2007; Song et al., 2005).

Viruses are known to alter cellular lipid metabolism to facilitate their own multiplication and, importantly, inhibition of cholesterol and fatty acid biosynthetic pathways has been shown to curtail virus replication, maturation and secretion (Clark et al., 2012; del Real et al., 2004; Munger et al., 2008; Peña and Harris, 2012; Potena et al., 2004; Spencer et al., 2011; Taylor et al., 2011; Ye et al., 2003; Yu et al., 2012). In this regard, interferon (IFN)-mediated downregulation of the sterol biosynthesis pathway has been shown to be an integral part of Mϕ host defense (Blanc et al., 2011; Watterson et al., 2012). It is well recognized that IFNs, in particular type I, play a pivotal role in inhibiting viral growth through the activation of IFN-stimulated genes (ISGs). However, the specific functions of many ISGs and the mechanisms by which they restrict viral replication are not well defined (O’Neill and Bowie, 2010). The notion that there is an intrinsic connection between the sterol network and IFN is further supported by studies showing IFN modulation of the cholesterol hydroxylase gene, *Ch25h* (Bauman et al., 2009; Diczfalusy et al., 2009; Park and Scott, 2010). Importantly, however, the precise mechanisms and physiological roles by which IFN regulates the sterol metabolic network remain unclear.

Here, we have undertaken a comprehensive, unbiased investigation of the biological role of oxysterols produced by Mϕ during infection and upon IFN stimulation. We provide evidence for a biological role for 25HC as a lipid effector of the innate immune response, highlighting the targeting of host metabolic regulatory networks during viral infections and, more broadly, inflammation.

## Results

### Quantitative Oxysterol Profiling of Infected or Interferon-Activated Primary Macrophages

**Figure 1:**
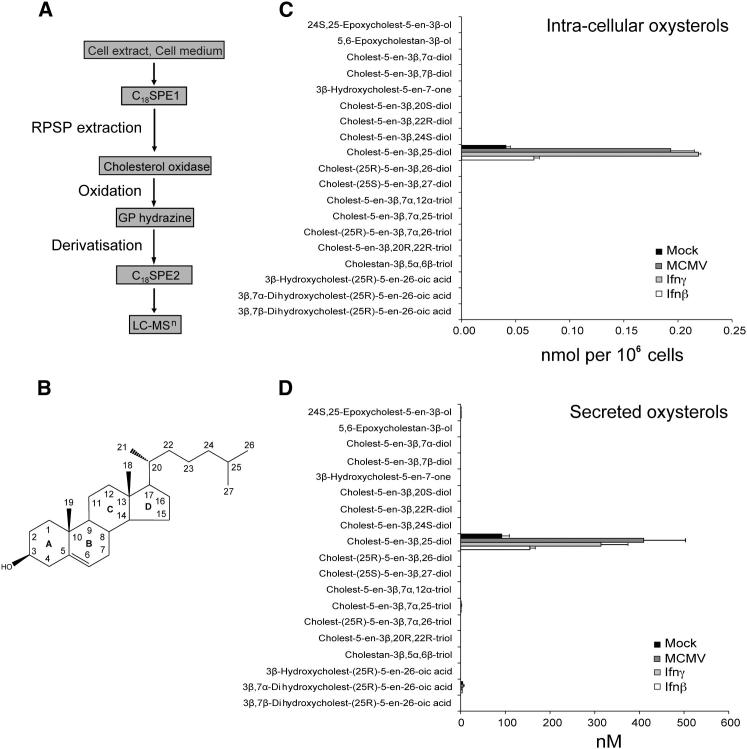
25HC Synthesis and Secretion by Mϕ after Interferon Treatment or MCMV Infection (A) Work-flow for the LC-MS(MS^n^) analysis of oxysterols. Quantification was performed from reconstructed ion chromatograms by reference to deuterated internal standards. (B) Structure of cholesterol, the scaffold on which oxysterols are built. (C) Intracellular levels of oxysterols in BMDM following MCMV infection (MOI = 1), Ifn-γ (25 U/ml) or Ifn-β (25 U/ml) stimulation for 12 hr. A mock sample of BMDM without stimulation or infection was similarly analyzed. (D) Analysis of oxysterols secreted by cells into medium when treated as in (C). n = 3, data are mean ± SEM.

We speculated that the synthesis of oxysterols might have a role in the negative regulation of sterol biosynthesis upon infection (Blanc et al., 2011). Accordingly, we performed a comprehensive quantitative determination of all known naturally occurring side-chain and steroid-ring oxysterol metabolites that are produced and/or secreted by either infected or interferon (β or γ) stimulated primary Mϕ (Figures 1A, 1C, and 1D). Bone-marrow-derived macrophages (BMDMs) were infected (mouse cytomegalovirus, MCMV) or stimulated (Ifn-β or Ifn-γ) for 12 hr and the oxysterol content of cells and medium measured by LC-MS(MS^n^) following derivatization (see Figure 1A for workflow schematic). By generating reconstructed ion chromatograms (RICs) for mono- and dihydroxycholesterols, epoxycholesterols, and mono- and dihydroxycholestenoic acids (see Figure 1B for chemical structure), essentially all naturally occurring oxysterols and their immediate downstream metabolites were profiled. Figure 1 shows the list for common oxysterols and those oxysterols not shown were inclusive for 3β-hydroxycholest-5-en-22-one, cholest-5-ene-3β,4β-diol, cholest-4-ene-3β,6α -diol, cholest-4-ene-3β,6β –diol, cholest-5-en-3β, 19-diol, cholest-5-ene-3β,22S-diol, cholest-5-ene-3β,24R-diol, cholest-5-ene-3β,7β,25-triol, cholest-(25R)-5-ene-3β,7β,26-triol, cholest-5-ene-3β,24,25-triol, 3β-hydroxycholest-(25S)-5-en-27-oic acid.

The results of this oxysterol-metabolome and secretome screen are summarized in Figure 1C (intracellular) and 1D (secreted). Interestingly, in this study noticeable amounts of 25HC were found in unstimulated Mϕ but not media—a result that perhaps reflects a pre-existing subprimed activation of Mϕ (Kropp et al., 2011). Strikingly, amounts of only 25HC were markedly increased by infection or treatment with MCMV or Ifn (β or γ), respectively (Figures 1C and 1D). In this study, the only other detectable oxysterols (with systemic names provided in brackets) were 24(S),25-epoxycholesterol (24S,25-epoxycholest-5-en-3β-ol), 3β,7α-dihydroxycholestenoic (3β,7α-dihydroxycholest-[25R]-5-en-26-oic) acid and 7α,25-dihydroxycholesterol (cholesten-5-en-3β,7α,25-diol) but at extremely low levels. Because LC-MS(MS^n^) assays are exceptionally sensitive and these products were detected in the media only, it is possible that they were generated by auto-oxidation. Notably, no other oxysterol or downstream 3-oxo-4-ene metabolites of the listed oxysterols were found. Similarly, we found no evidence for oxysterol mono- or disulphates. The sulphation of oxysterols is known to catabolically inactivate oxysterol activity and also generate alternative-biologically active products. However, we find no detectable presence of either the 3- or 25-monosulphate or 3,25-disulphate.

**Figure 2:**
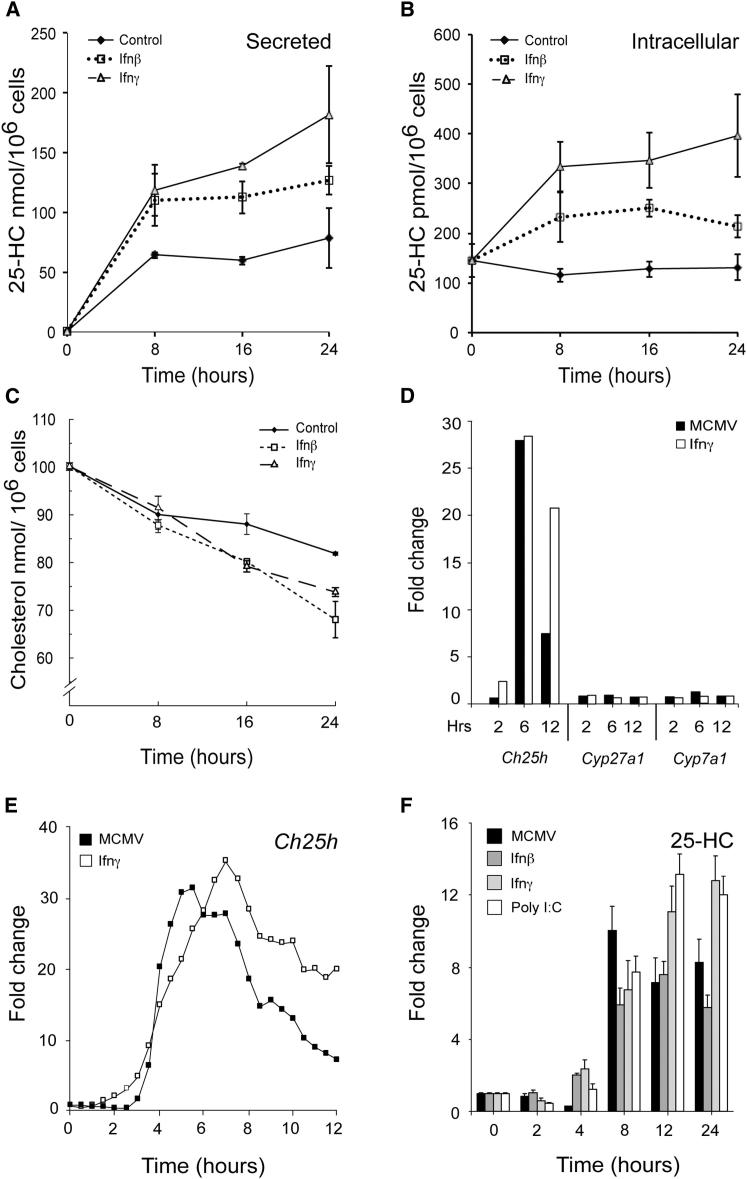
Temporal Analyses of *Ch25h* Synthesis and 25HC Production (A) Levels of secreted (A) and cellular (B) 25HC and cellular cholesterol (C) with Ifn-β (25 U/ml) or Ifn-γ (25 U/ml) in delipidized media. n = 3, mean ± SEM. (D) Expression level of *Ch25h*, *Cyp27a1*, and *Cyp7a1* from microarray time course experiment in WT BMDM following MCMV infection (MOI = 1) or Ifn-γ (10 U/ml) stimulation (*Cyp46a* and *Cyp7b1* did not reach the threshold of expression). (E) *Ch25h* expression BMDM following MCMV infection (MOI = 1) or Ifn-γ (10 U/ml) stimulation. (F) 25HC level in BMDM 24 hr following MCMV infection, Ifn-β (10 U/ml), Ifn-γ (10 U/ml) or PolyI:C (10 ng/ml) with the mean ± SEM for data obtained from three independent biological replicates.

To avoid potential issues relating to serum-derived lipid contamination, we proceeded to investigate the production of 25HC using lipid-depleted media. Time course experiments revealed that the majority of 25HC was secreted during the first 8 hr of incubation, and levels continued to rise more gradually until the 24 hr endpoint (Figures 2A and 2B). As previously reported for cells grown in normal media, a steady and significant fall in free cholesterol, and its direct precursor desmosterol, was observed with time suggesting a reduced biosynthetic output (Figure 2C; data not shown) (Watterson et al., 2012). In summary, the LC-MS(MS^n^) experiments revealed 25HC as the only oxysterol notably upregulated following macrophage infection or activation by IFNs. This occurred during the first hours after infection or treatment before overall levels of cholesterol in treated cells fell notably below those of the control. Figures 2D and 2E show data that suggests that the highly-specific increase in 25HC was attributable to upregulated expression of the hydroxylase *Ch25h*; levels of the only other expressed hydroxylases *Cyp27a* and *Cyp7a* that are also implicated in 25HC metabolism were unaltered. When a different perspective on this result was sought and interferon or polyI:C was used to stimulate BMDMs, increased levels of secreted 25HC were clearly detectable 4 hr after treatment. PolyI:C is a potent Tlr3 agonist that stimulates high levels of Ifn-β production (Figure 2F). Notably, in contrast to the observed decline in *Ch25h* transcript expression, levels of secreted 25HC metabolite levels remained relatively high until 24 hr in these experiments. We conclude from the above data that interferon activation or MCMV infection of Mϕ results in a large and highly focused production of intracellular and secreted 25HC.

### Exogenous Treatment and Endogenous Secretion of 25HC by BMDM Has Broad Antiviral Potency

**Figure 3:**
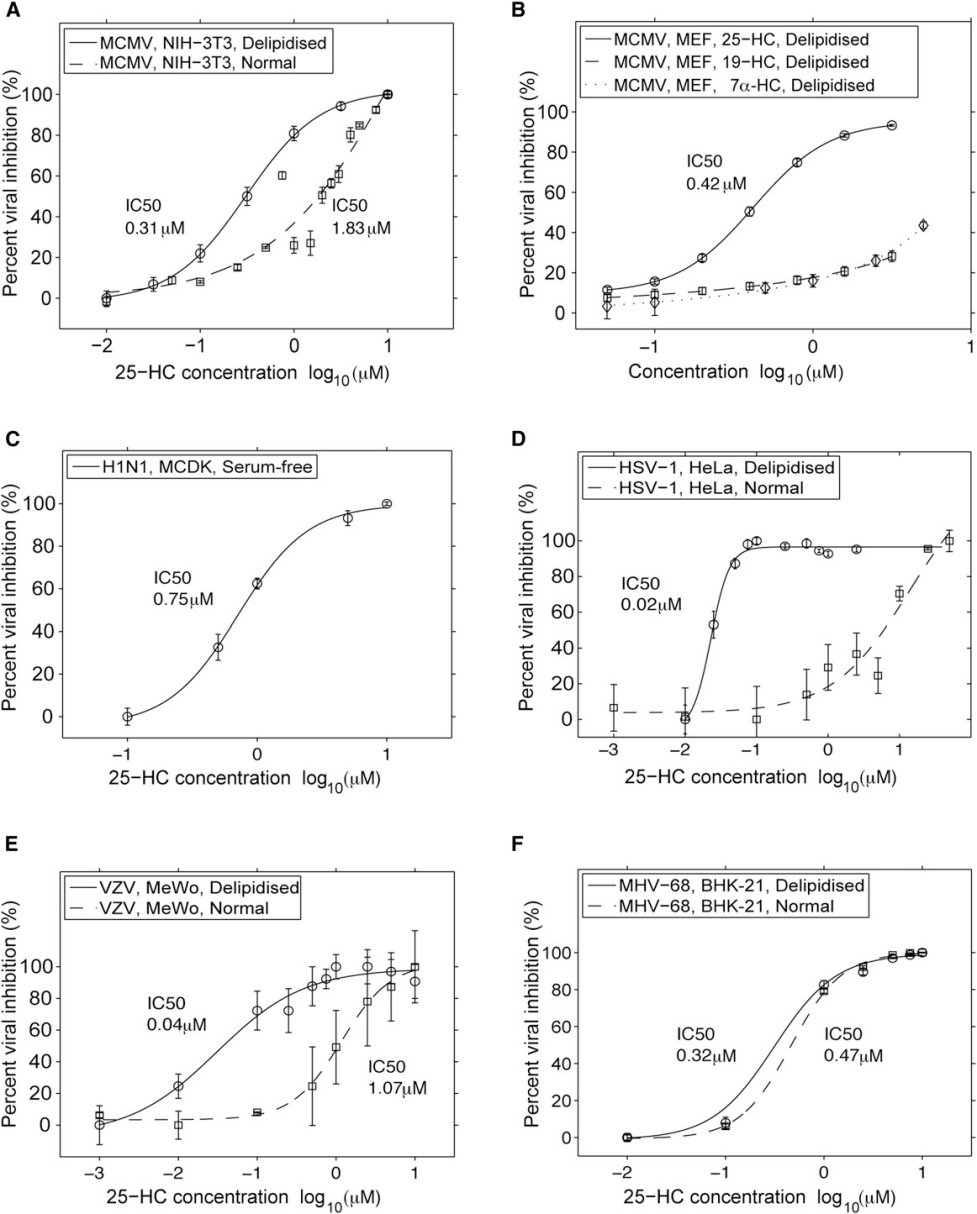
Analysis of 25HC Anti-Viral Effects, See Also Figure S1 Virus replication was monitored (A, B, D, E, and F) as a function of kinetic GFP fluorescence. Replication slopes over the linear phase were calculated and normalized to vehicle treated cells, and the mean replication slope from independent experiments calculated. For (C) Influenza virus, supernatant was collected at 16 hpi for analysis of viral titer by plaque assay. Data are mean ± SEM (three replicates).

It is possible that 25HC might function as an antiviral mediator in the IFN-regulated sterol metabolic network (Blanc et al., 2011). To test this idea, we sought to address whether physiologically relevant concentrations of 25HC, secreted by activated Mϕ, are capable of conferring an antiviral state. We first determined in dose-response experiments the inhibitory concentrations (IC) for viral growth of MCMV in normal serum (i.e., sterol-replete media would favor restricted Srebp2 processing). Figure 3A shows that under these conditions 25HC has an IC_10_ of approximately 0.1 μM and an IC_50_ of 2 μM and did not induce a cytotoxic effect (see Figure S1 available online). Because this level of inhibition is greater than the known levels of 25HC that are sufficient to disrupt Srebp processing (Radhakrishnan et al., 2007), we infer from these experiments that in sterol-replete conditions 25HC is unlikely to involve the Srebp processing pathway for maximal antiviral activity (Adams et al., 2004; Yabe et al., 2002).

Because cells grown under lipid-deficient media conditions have an increased dependency on sterol biosynthesis, we also evaluated the antiviral effect of 25HC under lipid-depleted conditions (Sun et al., 2007). As shown in Figures 3A and 3B, there is an increase in sensitivity of MCMV to inhibition by 25HC under these conditions. This antiviral effect is specific to 25HC because other related enzymatically generated oxysterols, 19-HC and 7α-HC, fail to repress viral infection (Figure 3B). These oxysterols are known not to inhibit the Srebp-pathway (Radhakrishnan et al., 2007). In contrast, other oxysterols known to inhibit the Srebp-pathway through INSIG, such as 27-HC and 24(S), 25 epoxycholesterol, are similarly capable of inhibiting MCMV growth (Figure S1B). These observations point to the possibility that although the Srebp pathway is not absolutely essential for mediating the antiviral effects of 25HC, it might contribute toward enhancing the sensitivity to 25HC inhibition. The physiological relevance for the lipid dependency is presently unclear. The differential sensitivities under sterol-replete and -depleted conditions raise the possibility for multiple levels of control. To determine whether the differential modes of inhibition by 25HC are specific to MCMV or whether it might have a broader antiviral role, we next evaluated, in normal or lipid-depleted conditions, its potency to inhibit a range of other viruses in different cell systems. Figures 3C–3F show the quantitative determination of the dose-dependent potency of 25HC to inhibit Influenza A (H1N1), herpes simplex virus-1 (HSV-1), varicellar zoster virus (VZV) and murine gamma herpes virus 68 (MHV-68) viral infections in MDCK, HeLa, MeWo, and BHK cells, respectively. The results of these titration experiments clearly show a broad and highly potent (IC_50_ values ranging from 20 nM to 750 nM) antiviral role of 25HC (Figures 3D–3F). This effect is selective because Figure S1C shows that under the conditions tested, 25HC does not specifically inhibit Adenovirus 5 or 19a. We conclude that 25HC has a high potency to inhibit a broad spectrum of viruses from high to low physiological concentrations depending on lipid conditions and virus-host cell system.

**Figure 4:**
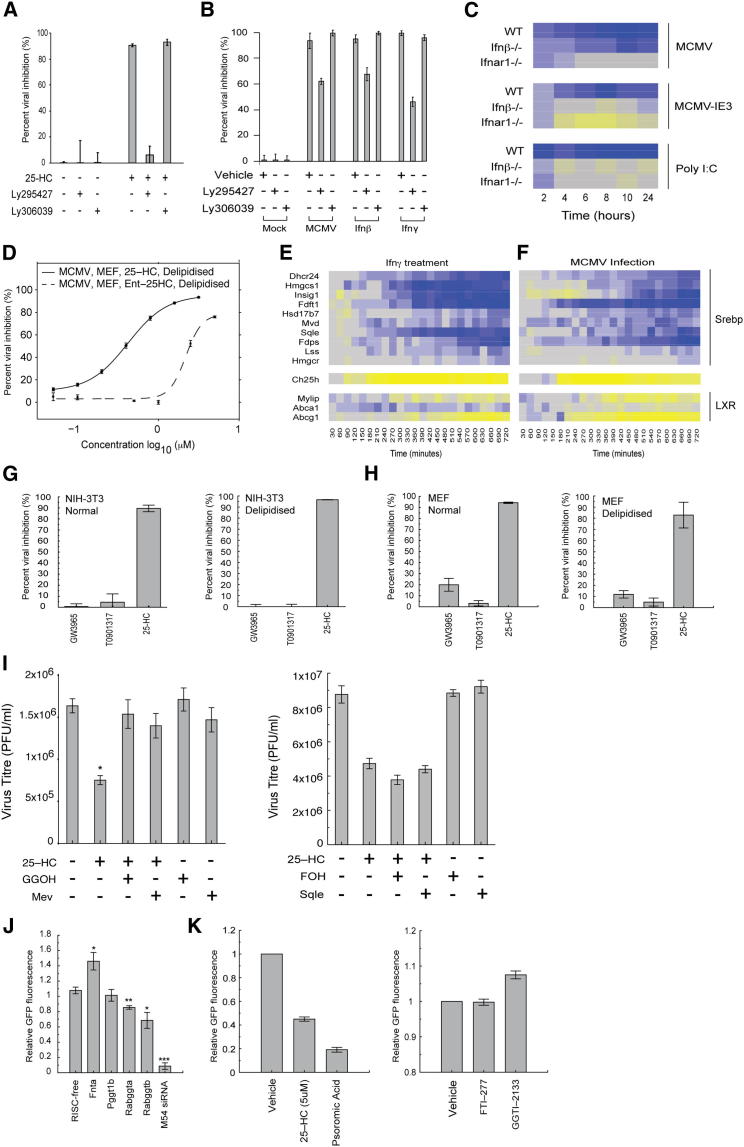
Investigation of Specificity and Selectivity of 25HC Antiviral Activity, See Also Figure S2 (A) NIH 3T3 cells were infected with MCMV-GFP virus (MOI = 0.05) treated with 25HC in the presence or absence of Ly295427 (20 μM) or Ly306039 (20 μM). The level of infection was measured by determining the GFP fluorescence level after 72 hr infection. n = 3, data are mean ± SEM (three replicates per experiment). (B) NIH 3T3 cells infected with MCMV-GFP virus (MOI = 0.05) received conditioned medium from BMDM infected, or treated with IFN-β (25 U/ml) or IFN-γ (10 U/ml) ± 20 μM Ly295427 or 20 μM Ly306039. n = 3, and data are mean ± SEM (with three biological replicates per experiment). (C) Expression analysis of *Gpr183* (relative to t = 0) in WT, *Ifnb*^*−/−*^ or *Ifnar1*^*−/−*^ BMDM infected with MCMV or MCMVdie3 (MOI = 1) or treated with PolyI:C (10 ng/ml). Each column represents one time point and row represents one gene. Gene expression is shown as a pseudocolor – blue = decrease, yellow = increase. Log fold change values were calculated by subtracting the t = 0 signal value from the signal value at time points after infection or treatment. (D) MEF were pretreated with 25HC or ent-25HC in delipidized medium for 24 hr and infected with MCMV-GFP (MOI = 1). n = 2, data are mean ± SEM (with three biological replicates per experiment). (E) BMDM treated with 10 U/ml Ifn-γ or mock-treated (F). Heatmap showing temporal alterations in abundance of sterol-related transcripts in Ifn-γ-treated BMDM (E, relative to t = 0) or MCMV infected BMDM (F, relative to t = 0) as in (C). (G) NIH 3T3 cells were pretreated with GW3965 (1 μM), T0901317 (1 μM) or 25HC (10 μM) in normal or delipidized medium for 24 hr and then infected with MCMV-GFP. After infection, all wells received fresh medium containing normal or delipidized serum and GW3975, T0901317, or 25HC. Data are normalized to negative controls treated with vehicle alone and are the mean of six independent experiments ± SEM (three biological replicates per experiment). (H) MEFs were pretreated with GW3965 (1 μM), T0901317 (1 μM) or 25HC (10 μM) in normal or delipidized medium for 24 hr and then infected with MCMV-GFP as described in (G). Data are normalized to negative controls treated with vehicle alone and are the mean of six independent experiments ± SEM (three replicates per experiment). (I) Left shows MEFs infected with MCMV (MOI = 0.1) incubated with 25HC (1 μM) and/or GGOH (20 μM) or MEV (20 μM) and MCMV titer determined by plaque assay. Data are mean of three independent experiments ± SEM. ^∗^p ≤ 0.05. Right shows that MEFs infected with MCMV (MOI = 0.1) were incubated with vehicle, 25HC – (1 μM), and/ or Farnesol (FOH – 20 μM) or Squalene (Sqle – 20 μM) and MCMV titer was determined by plaque assay. Data are the mean of two independent experiments. (J) RISC-Free, Fnta, Fntb, Pggt1b, Rabggta, Rabggtb, or MCMV M54 siRNA (25nM) were reverse-transfected into NIH/3T3 cells. Forty-eight hr after transfection, cells were infected with MCMV-GFP (MOI = 0.05) and virus replication monitored. Data are normalized to RISC-free wells and are mean of six independent experiments ± SEM.^∗^p < 0.05, ^∗∗^p < 0.01, ^∗∗∗^ < 0.001. (K) Left shows that MEF were pretreated with 25HC (5 μM) or psoromic acid (20 μM) for 24 hr, infected with MCMV-GFP (MOI = 0.05), and then incubated with EMEM with the same concentrations of 25HC or psoromic acid. Data are normalized to vehicle treated wells and are mean of two independent experiments. Right shows that MEFs were pretreated with vehicle, FTI-277 (10 μM), or GGTI-2133 (10 μM) for 24 hr, infected with MCMV-GFP (MOI = 0.05), and incubated with the same concentrations of FTI-277 or GGTI-2133 as prior to infection. Data are normalized to vehicle treated wells and are mean of two independent experiments with three biological replicas (error bars represent range).

The pharmacologic agent LY295427 (3α,4α,5α)-4-(2-propenylcholestan-3-ol) is a known antagonist of 25HC, whereas LY306039, the 3β-isomer of LY295427, fails to derepress the effect of 25HC (Janowski et al., 2001). We used these agents to test whether they have any efficacy in interfering with the antiviral activity mediated by 25HC. Figure 4A shows the ability of exogenous 25HC to potently inhibit viral growth and that the observed antiviral effect can be completely blocked in the presence of LY295427 but not LY306039. These results validated our subsequent use of LY295427 in interfering with endogenously secreted 25HC. Accordingly, conditioned media (10 hr posttreatment) from mock-treated, infected, Ifn-β, or Ifnγ-treated Mϕ was incubated with vehicle alone, or with LY295427 or LY306039 and then applied to MCMV-infected NIH 3T3 cells. As expected, mock-conditioned media failed to inhibit viral growth and drug treatment had no effect (Figure 4B). Meanwhile, conditioned media from infected or IFN-treated cells had pronounced antiviral activity. In the presence of LY295427, antiviral activity was partially blocked (Figure 4B). We interpret the results of these experiments to show that endogenously secreted 25HC has a potent antiviral activity and, if we assume, LY295427 is an exclusive inhibitor of 25HC, this accounts for at least 40%–50% of the antiviral secretome activity. It is likely that the secretion of other antiviral cytokines rather than an incomplete blockade provides the remaining antiviral activity. Overall, these experiments demonstrate that Mϕ activated *Ch25h* generates biological antiviral activity through paracrine, and in the case of Mϕ also autocrine effects of 25HC.

### 25HC Antiviral Actions Are LXR Independent and Enantioselective

The above studies raise the possibility for potentially direct and indirect modes of 25HC action. It is conceivable that inhibitory effects of 25HC above 0.5 μM might involve an indirect membrane-mediated mechanism, whereas those below 0.5 μM may have a direct protein mediated mode of action. It is also plausible that 25HC could be metabolized to a more potent antiviral metabolite. A candidate pathway is the Epstein-Barr virus-induced gene 2 (*EBI2/GPR183*) enzyme system (Hannedouche et al., 2011; Liu et al., 2011). The natural ligand for EBI2 has been identified as 7α,25-diHC. In this pathway, a critical enzyme required for the generation of 7α,25-diHC is CH25H, however, in our mouse experiments *Ebi2* gene expression is downregulated in Mϕ upon productive and nonproductive infection as well as polyI:C treatment, in an IFN-dependent manner (Figure 4C), suggesting that it is unlikely to be involved in the antiviral response. Intriguingly, the coordinated IFN suppression of the *Ebi2* mediated 25HC-catabolic-pathway further underscores the highly focused 25HC Mϕ response and is unlikely to involve an intermediary metabolite mode of action.

To evaluate generalized membrane effects and further test the specificity of 25HC actions, we studied the activity of *ent*-25HC, an enantiomer of 25HC. In these experiments, we assumed the well-known ability of 25HC to interact and change the properties of cellular membranes would be enantiomer insensitive while protein specific inhibitory effects on viral growth would be restricted to 25HC. Figure 4D shows that *ent*-25HC has antiviral activity but only at concentrations exceeding 1 μM, which is below the nonselective effects of oxysterols on viral infection and is an order of magnitude less potent at inhibiting MCMV than 25HC (Figure 4D). These results show that the effect of 25HC on antiviral activity is highly enantio-selective, consistent with the idea that 25HC antiviral functions occurs through a specific protein target(s). Because *ent*-25HC can exert an antiviral effect at high concentrations it is likely that this activity occurs via an indirect mode of action mediated at the membrane level. Notably, the antiviral effect of *ent*-25HC provides evidence for Srebp-independent antiviral pathway that might act as a cofactor for membrane proteins or acting as a determinant for membrane lipid composition or compartmentalization (Contreras et al., 2012).

In the case of lipid-depleted conditions, our data is consistent with a subordinate inhibition of the Srebp pathway by 25HC and by other oxysterols such as 24S, 25-epoxycholesterol or 27-HC. Notably, these oxysterols are also known to activate LXR (Janowski et al., 2001). Thus, we next evaluated the transcriptional responses of LXR and Srebp target genes in Ifnγ-treated or MCMV-infected Mϕ. In these experiments, molecular profiling of Ifnγ and MCMV infected Mϕ every 30 min for 12 hr showed the expected downregulation of Srebp2 target messenger RNAs (mRNAs) including *Insig1* (Figure 4E). However, the temporal dynamic changes were complex. In this analysis, increased expression of *Ch25h* mRNA levels is associated with a subsequent activation of the LXR target genes *Mylip* and *Abcg1*. To directly test whether LXR ligand activation is sufficient to develop an antiviral state, we treated MCMV infected cells with 1 μM of the potent synthetic LXR ligands (GW3965 and T0901317). Although these ligands were active in LXR-dependent reporter assays (Figure S2A), they failed to elicit an antiviral response and, therefore, we conclude that LXR activation is insufficient to affect viral inhibition of MCMV in both normal or lipid depleted media conditions (Figures 4G and 4H; Figures S2B and S2C).

### 25HC Antiviral Action Partially Involves the Mevalonate Branch, Blocking Postentry Viral Growth

A decrease in the Srebp2 target genes of the sterol pathway and especially the mevalonate-isoprenoid side branch is notable (in particular *Mvd* and *Fdps*) (Figures 4E and 4F). Mevalonate is a shared precursor of coenzyme Q, ubiquinone, dolichol, farnesyl, and geranylgeranyl pyrophosphates. The latter two isoprenoids are necessary for protein prenylation. Many viruses require prenylation of essential viral or host proteins (e.g., the large delta antigen of HDV or the pseudorabies virus tegument protein) (Bordier et al., 2003; Clase et al., 2003) or (e.g., host protein FBL2 for hepatitis C virus [HCV] or RhoA activation for RSV) (Ye et al., 2003) (Wang et al., 2005) (Gower and Graham, 2001). In the case of MCMV, we find, upon inhibition of the prenyltransferase branch by RNAi, that viral replication is selectively reduced upon knockdown of GGTase II but not GGTase I or farnesyltransferase (Figure 4J), confirming previous work defining the dependency of MCMV for the isoprenoid pathway (Blanc et al., 2011). In the present study, Rabggta and Rabggtb small interfering RNA (siRNA) reduced the abundance of their target transcript by around 60% (Figure S4F) and none of the above siRNA significantly reduced cell viability (Figure S4D). To investigate whether 25HC antiviral functions involve the mevalonate branch, we treated MCMV infected cells with nonsaturating inhibitory levels of 25HC (<1 μM) and coincubated with mevalonolactone (MEV), geranylgeraniol (GGOH), or Farnesol (FOH), and the amount of infectious virus determined. In these metabolic rescue experiments, viral growth was restored with MEV or GGOH but not FOH (Figure 4I). However, in the presence of saturating inhibitory concentrations of 25HC (>5 μM), viral growth is not recovered with MEV, GGOH, or FOH cotreatment (data not shown) indicating that under these conditions the isoprenoid-prenylation arm is not required. It is possible that other derivatives such as dolichol may be required. Altogether, these investigations are indicative that for MCMV, 25HC blocking of the mevalonate branch imparts a contributory role but is not absolutely essential. As expected, inhibitors of GGTase I (GGTT-2133) or farnesyltransferases (FTI-277) fail to inhibit MCMV, whereas psoromic acid (an inhibitor of GGTase II [Deraeve et al., 2012]) restricts viral growth (Figure 4K). None of these compounds significantly affected cell viability (Figure S2E).

**Figure 5:**
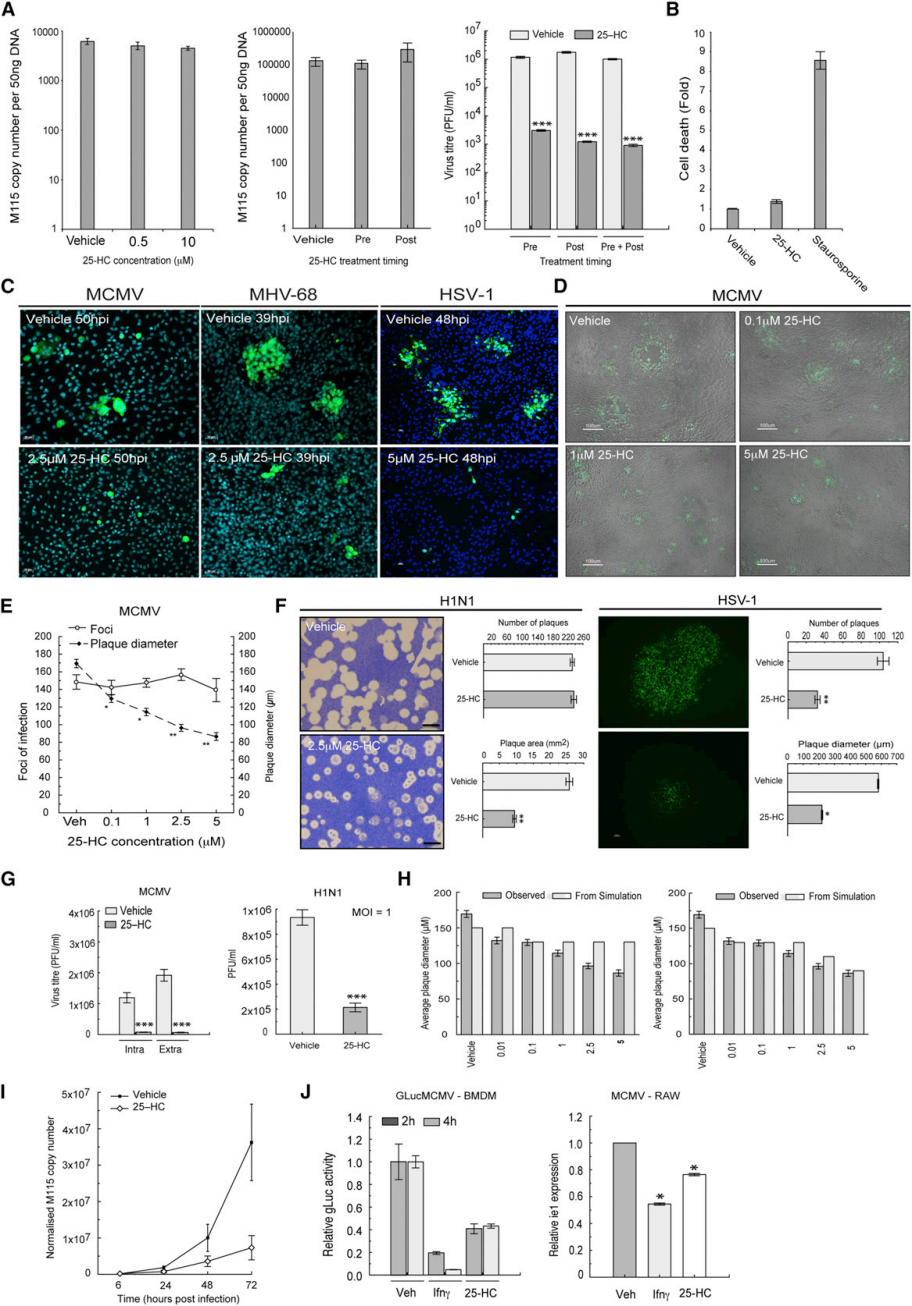
Analysis of 25HC Effects on Cell Death, Virus Entry and Cell-to-Cell Spread, See Also Figure S3 and Movie S1 (A) Left shows NIH 3T3 cells treated with 25HC in delipidized medium and MCMV infected (MOI = 2). Three hpi DNA levels were determined by qRT-PCR. Middle shows RAW 264.7 cells treated with 25HC (5 μM) pre- or postinfection with MCMV (MOI = 1) in normal medium. At 3 hpi, amounts of internalized viral DNA were determined by qRT-PCR. Right shows MEFs treated with 25HC (5 μM) 24 hr prior to and after infection with MCMV (MOI = 2.5) in delipidized media and infected virus titers determined by plaque assay 4 dpi. n = 4, with mean ± SEM. ^∗∗∗^p ≤ 0.001. (B) NIH 3T3 cells were treated with 10 μM 25HC or 10 μM staurosporine in normal media and cell death measured. Data are mean ± SEM for three replicates. (C) Left shows images of NIH 3T3 cells with and without 25HC 2.5 μM infected with MCMV (MOI 0.01). Middle shows cells treated with 2.5 μM 25HC in normal media. Images of NIH 3T3 cells infected with MHV-68 39 hpi. Right shows HeLa cells infected with HSV-1 VP26-YFP (MOI = 3), treated with 25HC (20 μM) in normal medium and images taken 24 hpi. (D) NIH 3T3 cells infected with MCMV (MOI = 0.005) and treated with 0.1, 1, or 5 μM 25HC in normal media. Images were taken at 72 hpi. (E) *p53*^−/−^ MEFs were infected with MCMV-GFP (MOI = 0.001) and overlaid in normal medium containing 2.5% agarose and 0.1, 1, 2.5, or 5 μM 25HC. Plaque diameter was measured 3 dpi. Data are mean 170 and 220 plaques counted ± SEM (three biological replicates). ^∗^p ≤ 0.05, ^∗∗^p ≤ 0.01. (F) H1N1: Plaque reduction assay of Influenza A/WSN/33 (H1N1) virus infection of MDCK cells in the presence or absence of 25HC (2.5 μM) and a 0.5% agarose overlay. Forty-eight hpi, cells were stained with 0.1% toluidine blue and number and size of plaques determined. Left upper (vehicle) and lower (25HC) panels are representative images showing differences in plaque size and morphology. Right panels show mean number of plaques per well (upper) and mean plaque area (lower, mm^2^) per well. HSV-1-eGFP (C12) infection of A549 cells pretreated with vehicle or 25HC (5 μM) with a 0.5% agarose overlay. Plaque numbers and diameters were determined 72 hpi. cell. Representative fluorescent microscopic images of plaques from vehicle- (top left) or 25HC- (bottom left) treated cells are shown. Plaque numbers (top right) and mean plaque diameter (bottom right) from HSV-1 infected cells are shown. n = 6, data are mean ± SEM. ^∗^p ≤ 0.05, ^∗∗^p ≤ 0.01. (G) Left shows that NIH 3T3 cells were infected with MCMV (MOI = 2.5) in delipidized medium containing vehicle or 25HC (5 μM). Two dpi supernatants (extracellular virus) and intracellular virus quantitated by plaque assay. n = 4, mean ± SEM. ^∗∗∗^p ≤ 0.001. Right shows MDCK cells infected with A/WSN/33 (H1N1) influenza virus (MOI = 1), serum-free medium containing vehicle, or 25HC (2.5μM), and 0.5% agarose was added to the monolayers. After 16 hpi, monolayers were fixed and stained. n = 4 mean ± SEM. ^∗∗∗^p ≤ 0.001. (H) Left shows plaque diameters observed in experiment and predicted by modeling when cell permissivity and viral growth both mediate 25HC antiviral effect. Right shows that plaque diameters observed in experiment and predicted by modeling when only cell permissivity contributes to the infectious transmission between cells in a dose-dependent manner. (I) MEFs pretreated with 25HC (5 μM) for 24 hr, infected with MCMV (MOI = 0.3), and DNA levels quantitated at indicated times. (J) Left shows that BMDM were pretreated with Ifn-γ (10 U/ml) or 25HC (5 μM) for 24 hr, infected with GLucMCMV and at 2 or 4 hpi and Gaussia luciferase activity measured using supernatants. n = 6 ± SEM. Right shows that RAW264.7 cells were pretreated for 24 hr with Ifn-γ (10 U/ml) or 25HC (2 μM), infected with MCMV (MOI = 2), in normal medium containing vehicle, IFN-γ (10 U/ml) or 25HC (2 μM) for 24 hr, relative abundance of the MCMV ie1 assayed by qRT-PCR. n = 3; mean ± SEM. ^∗^p ≤ 0.05.

We next investigated at what stage in infection 25HC blocks MCMV growth. The results shown in Figure 5A indicate that the level of internalized MCMV genomes is unaffected by 25HC. Furthermore, in plaque reduction assays the number of primary infectious foci is equivalent in the presence or absence of 25HC (Figure 5E). These results argue against viral entry as a primary mode for MCMV inhibition. In this connection, we also find that 25HC is highly effective at blocking (at 40 nM inhibitory concentration) VZV infection of MeWo cells (Figure 3E). This represents an infection model in which cell-free virus is not produced and the infection process is exclusively dependent on cell-cell spread. 25HC at high concentrations has been shown to promote apoptosis, a well-characterized antiviral mechanism, in a range of cell types including Mϕ (Rusiñol et al., 2004). Notably, however, as shown for NIH 3T3 cells (Figure 5B), 25HC at high antiviral concentrations (10 μM) fails to significantly induce cellular apoptosis in comparison with staurosporine. Again, we cannot rule out that other cell-virus systems may employ a 25HC mediated apoptotic antiviral response. In this regard, we have found BMDM to be especially sensitive to 25HC concentrations above 2 μM.

We noted marked inhibitory effects on plaque development for all viruses investigated (Figure 5C–5G). Dose response analyses of MCMV plaque formation clearly indicated that the ability of the virus to spread decreased with increasing dose of 25HC (Figures 5C and 5D), while no statistical differences in numbers of infection foci in the presence or absence of 25HC were obtained (Figure 5E). In plaque reduction assays a significant dose-dependent difference in the diameter of the viral plaque was observed (Figure 5E). Similar results were also obtained from plaque reduction assays for influenza virus (Figure 5F). A simple mathematical model that best fits the experimental data is the one in which growth dominates over susceptibility to infection (Figure 5H; Figure S3F; Movie S1). In agreement, single hit growth analyses of MCMV and influenza infection show that 25HC effectively inhibits viral growth (Figure 5G) and for MCMV, 25HC inhibits yields of intracellular and secreted virus to the same extent (Figure 5G). However, in the case of HSV-1 we observed a significant difference in both plaque size and number of plaques (Figure 5F; Figure S3E) suggesting viral entry and growth can be affected and further indicating the multiple levels of control exerted by 25HC depending on the virus-host cell system.

In accordance with the above results we observed a reduction at the level of viral DNA replication by 25HC (Figure 5I). This is associated with reduced amounts of early (E1) and late (M115) gene expression in a 25HC dose-dependent manner strongly supporting a postentry stage inhibition of MCMV infection (Figure S3A). For MCMV Mϕ infection, IFNs mediate antiviral effects through inhibition of the major immediate-early promoter (MIEP) (Kropp et al., 2011). We also find that 25HC imparts, in a dose dependent manner, inhibition of the MIEP monitored by using either reporter virus assay or the endogenous *ie1* expression levels, resulting in approximately 50% inhibitory activity in comparison with Ifnγ treatment (Figure 5J; Figures S3B and S3C).

### Interferon Stimulation of 25HC Production Involves Direct Binding of Stat1 to *Ch25h* Promoter

**Figure 6:**
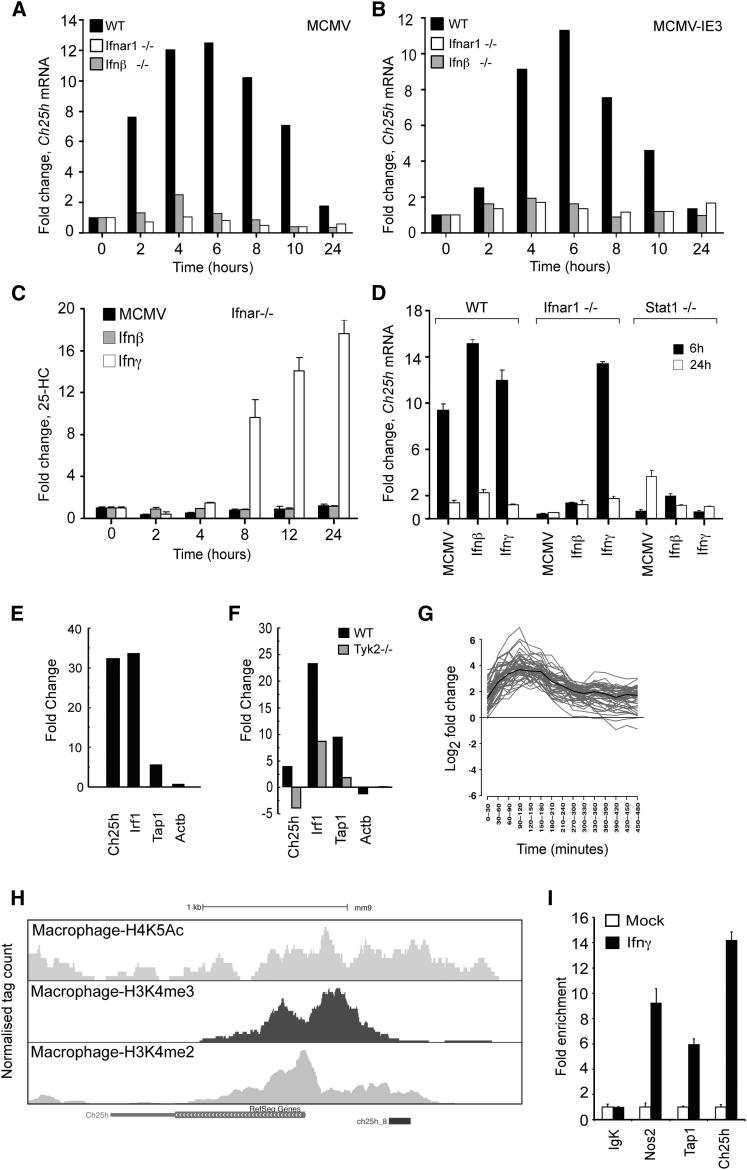
25HC Synthesis Is Interferon-Dependent and Regulated by Stat1; See Also Figure S4 and Tables S1 and S2 (A and B) *Ch25h* gene expression (relative to mock infected cells) in WT, *Ifnb*^*−/−*^ or *Ifnar1*^*−/−*^ BMDM infected with MCMV or MCMVdIE3 (MOI = 1). (C) Changes in intracellular 25HC in Ifnar1 KO BMDM following MCMV infection, Ifn-β (10 U/ml) or Ifn-γ (10 U/ml) stimulation for 24 hr. n = 3; data are mean ± SD. (D) qRT-PCR analysis of *Ch25h* mRNA expression at 6 and 24 hpi with MCMV (MOI = 1) or treatment with Ifn-γ (10 U/ml) or Ifn-β (25 U/ml) in WT, *Ifnar1*^*−/−*^ or *Stat1*^*−/−*^ BMDM. n = 3, data are mean ± SD. (E) Differential ntRNA synthesis between 60 and 90 min in Ifn-γ treated (F) or MCMV-infected BMDM (H) relative to mock were analyzed and fold-change values in treated or infected cells calculated relative to the control. (G) WT BMDM treated with Ifn-γ (10 U/ml). Microarray analysis of newly transcribed RNA isolated from 16 successive 30 min windows after treatment. (H) UCSC genome browser image is shown for *Ch25h* locus indicating Stat1 binding site (labeled ch25h_8) in promoter region. Normalized tag counts for H3K4me2-MNase ChIP-Seq, H4K5Ac-ChIP-Seq, and H3K4me3-ChIP-Seq are shown for untreated BMDM. (I) ChIP analysis of Stat1 binding at *Igk* (negative control), *Tap1*, and *Nos2* (positive controls), and *Ch25h* loci (as indicated in Figure 5I) in mock- or Ifn-γ− (20 U/ml, 1 hr) treated BMDMs. Fold enrichment for each locus is the ratio of percentage input of Ifn-γ-treated versus mock-treated BMDMs. IgK (negative control) refers to genomic region lacking Stat1 enrichment. Data are representative of two independent experiments performed in duplicate.

A central question regarding the role of 25HC in terms of innate immunity is how IFN signaling precisely regulates *Ch25h*. Thus, in our next experiments we sought to investigate in further detail the regulatory interferon dependence of the 25HC responses by using host and viral genetic knockouts in our macrophage-infection experiments. First we assessed whether the IFN-β plays a role in mediating the induction 25HC in infection. Figure 6A shows that greater than 80% of the induction response of *Ch25h* is lost in *Ifnb1*^*−/−*^ Mϕ, whereas 100% of the response is eliminated in *Ifnar1*^*−/−*^ Mϕ. Because all type I IFNs signal through the Ifnar1 receptor, these results indicate that upon infection of Mϕ, Ifn-β is the primary mediator, whereas other members of the type 1 interferon family serve a minor nonredundant role.

To determine whether a productive infection is required, we used a mutant virus (MCMVd*ie3*) that is fully proficient in infecting cells but fails to initiate viral gene expression. In the following experiments, infection of Mϕ with MCMVd*ie3* resulted in an equal response of *Ch25h* activation in comparison with the parental wild-type (WT) virus but with slightly delayed temporal kinetics (Figure 6B). In this case, and in comparison with *Ifnar1*^*−/−*^-infected cells, Ifn-β dominates the response (Figure 6B). These results show that productive infection is not required; indicating that pattern recognition receptor activation by the viral particle is sufficient to activate the transcriptional induction of *Ch25h*.

In our Mϕ cultures, Ifn-γ is not expressed but, as shown in Figure 1, is a potent inducer of *Ch25h* activity. Figure 6C shows that infection or treatment with exogenous Ifn-β of *Ifnar1*^*−/−*^ Mϕ abolishes the induced levels of 25HC, whereas exogenous Ifn-γ treatment does not. Because signal transducer and activator of transcription factor 1 (Stat1) is activated by Ifn-γ though its receptor Ifngr and Stat1 and Stat2 are activated by Ifn-β through its receptor Ifnar1, we next tested the ability of MCMV, Ifn-β and Ifn-γ to activate *Ch25h* mRNA levels in *Stat1*^*−/−*^ cells. Figure 6D clearly shows that genetic elimination of Stat1 results in the marked loss of the ability of Mϕ to activate *Ch25h* upon infection or treatment with type 1 or type 2 IFNs.

To further investigate the kinetic transcriptional induction of *Ch25h*, we quantitatively determined the de novo synthesis of RNA using 4-thiouridine incorporation. A schematic of this procedure is shown in Figure S4A. In these experiments, between 60 and 90 min after treatment, de novo synthesis of the *Ch25h* transcript increased by approximately 30-fold in Ifn-γ treated cells relative to the mock sample (Figure 6E). To test whether signaling from Ifnar1 was necessary for the induction of *Ch25h* during viral infection, we next conducted de novo synthesis experiments in *Tyk2*^−/−^ macrophage cells. The results shown in Figure 6F indicate that direct signaling by the receptor rather than an indirect or secondary mode of activation is crucial in virus-induced *Ch25h* synthesis and is consistent with the possibility of Stat1 involvement in this process. To explore this possibility further, we next conducted a microarray analysis of de novo transcription during the first 8 hr after IFN-γ stimulation of macrophages. An unbiased clustering analysis of temporal differential synthesis profiles identified a subset of 51 gene transcripts kinetically related to *Ch25h*, including Irf1 and Tap1, (Table S1; Figure 6G). Having demonstrated a notable increase in *Ch25h* transcript synthesis during the first 30 min after IFN-γ treatment, we sought to bioinformatically investigate promoter regions for common transcription factor binding sites. To this end, we applied a statistical analysis of transcription factor overrepresentation in transcripts increased by >2-fold during the first 30 min after IFN-γ treatment. An analysis of 109 transcripts identified (including *Ch25h*) (Table S2) revealed Stat1 as the only transcription factor identified with a combined *Z* score of >10 and Fisher score of <0.01 (values defined by empirical studies).

Because Stat1 is activated under all three conditions tested (MCMV infection, Ifn-β and Ifn-γ treatment) and multiple potential Stat1 binding sites are present upstream of *Ch25h* start site (Figures S4B and S4D). We sought to directly test these predictions using a chromatin immunoprecipitation (ChIP) of Stat1-bound DNA from BMDM treated with Ifn-γ for 1 hr (Figure 6I). The ChIP analysis of *Ch25h* promoter proximal sequences revealed an Ifn-γ-mediated recruitment of Stat1 to the predicted binding region of the *Ch25h* locus (Figure 6I). To extend this analysis, a ChIP-Seq analysis of histone modifications identified as markers of accessible and active chromatin regions, revealed that, in BMDM, the Stat1 binding occurs within a region characterized by these active signatures (Figure 6H). Collectively, these experiments show that *Ch25h* transcriptional activation is coupled to the interferon response through a direct molecular link with Stat1.

## Discussion

The innate immune response and the homeostatic pathways controlling cellular sterol levels are now known to regulate each other reciprocally (Castrillo et al., 2003; Kwak et al., 2000; Weitz-Schmidt et al., 2001). Our present findings demonstrate that Mϕ have evolved a program by which IFN is directly coupled to the regulation of 25HC. Although 25HC is a well-known negative feedback mediator of the sterol pathway, we present evidence that it can mediate, at multiple levels, antiviral cellular functions. We show that the mechanism for this coupling is through the direct recruitment of Stat1 to the promoter proximal region of the *Ch25h* gene. Strikingly, a comprehensive metabolome profiling of oxysterols shows that the classical activated Mϕ response is highly focused toward a single oxysterol, 25HC. We find that 25HC can impart antiviral cellular functions via an LXR-independent but Srebp-dependent subordinate mechanism, highlighting a previously unrecognized biological role for 25HC as part of the innate-immune response.

The subordinate involvement of the Srebp pathway for 25HC antiviral activity is based on the following: (1) In normal media, under conditions that reduce the cells reliance on the Srebp-processing pathway, IC_50_ values obtained are far higher than the known IC_50_ for 25HC inhibition of Srebp processing. (2) An enantiomer of 25HC inhibits viral infection; however, this only occurs at concentrations above 1 μM. We infer from this a Srebp-independent mode of action. (3) The antiviral potency of 25HC viral is dramatically increased for most, but not all, infections under lipid-depleted conditions that favor Srebp processing. Under these conditions, IC_50_ values for 25HC approximate those that actively inhibit the Srebp-pathway. (4) Oxysterols known to target the Srebp-pathway can effectively inhibit viral growth in lipid-depleted conditions, whereas those that do not regulate Srebp fail to affect viral growth. Future studies will be required to further understand the contribution of the Srebp-dependent and independent pathways to 25HC-induced antiviral activity.

How IFN couples with the regulation of the sterol metabolic network is relevant for both innate and adaptive immunity. In recent years, several groups have defined new mechanistic links between the sterol metabolic network and innate (Blanc et al., 2011; Diczfalusy et al., 2009; Im et al., 2011; Liu et al., 2012; Yi et al., 2012) or adaptive (Bauman et al., 2009; Hannedouche et al., 2011) immune responses. Similar to IFN, 25HC is found to halt MCMV growth in Mϕ cells by impeding postentry viral gene expression. IFN is known to dramatically alter in BMDMs both an increase and decrease in abundance of prenylated proteins (Vestal et al., 1995). One potential pathway for blocking MCMV growth is through altering the mevalonate (isoprenoid) branch of the pathway. Our previous studies had indicated that coincubation of IFN treated infected cells with GGOH partially rescues the antiviral response (Blanc et al., 2011). It is noteworthy that many viruses require prenylation of either viral or host proteins for productive infection (Amet et al., 2008; Bordier et al., 2003; Clase et al., 2003; Gower and Graham, 2001; Wang et al., 2005; Ye et al., 2003). Whether 25HC affects protein prenylation remains to be determined; however, we note that a number of highly specific prenylation inhibitors have been shown to significantly inhibit viral multiplication in vitro and in vivo (Bordier et al., 2003; Glenn et al., 1992; Gower and Graham, 2001; Ye et al., 2003).

In summary, our study demonstrates a previously unrecognized biological role for 25HC as an effector of the early innate-interferon response capable of imparting antiviral intracellular functions. Stat1 binding the *Ch25h* promoter provides a critical molecular link between innate immune stimulation, infection, and the Mϕ secretion of a single oxysterol, 25HC.

## Experimental Procedures

### Cell Propagation and Culture

BMDM were derived from femur and tibia isolated from C57BL/6 mice and grown in DMEM/F12 + GlutaMAX (Lonza, Vervier, Belgium) supplemented with 10% fetal bovine serum (FCS), 10% L929 conditioned medium (containing colony-stimulating factor Csf1) and Penicillin + Streptomycin. FACs assessed BMDMs for Mϕ cell surface markers, F480 and CD11b. All procedures were carried out under project and personal licences approved by the Secretary of State for the Home Office, under the United Kingdom's 1986 Animals (Scientific Procedures) Act and the Local Ethical Review Committee at Edinburgh University. All cultures are routinely tested for mycoplasma and endotoxin levels. Full details for these cultures and for MEFs and cell lines; NIH 3T3, RAW264.7, 199, MDCK, HeLa, MeWo and 293 cells are provided in the Supplemental Experimental Procedures.

### Reporter Viruses and Viral Plaque Assays

Viral growth including plaque assays, reporter GFP, and luciferase assays for viral growth and gene expression measurements for WT MCMV, MCMV-GFP, GLuc-MCMV Murine Gammaherpesvirus 68 (MHV-68-GFP), VZV-GFP (vaccine strain Oka), HSV-1-eGFP (C12), A/WSN/33 (H1N1) influenza virus, Ad5-gfp, and Ad19a-gfp are described in Supplemental Experimental Procedures. Plaque reduction assays used an agarose overlay for MCMV, HSV, and Influenza virus infection. For MCMV and HSV, plaque numbers and dimensions were quantified using fluorescent microscopy of infection foci. Phase-contrast and fluorescence visualization of infected was performed using a Zeiss Axio Observer Z1 inverted microscope (Carl Zeiss, Germany). Microscope control and image capture were undertaken using Axiovision Software (Carl Zeiss, Germany). All images were captured and diameters quantified using ImageJ software.

### Metabolic Profiling of Oxysterols

Details for the extraction of oxysterols and sterols are provided in Supplemental Experimental Procedures. The extracted oxysterol and sterol fractions were derivatized with Girard P reagent and analyzed by LC-MS(MS^n^) on a LTQ-Orbitrap Velos. Mass spectra were recorded at high resolution in the Orbitrap analyzer, and MS^n^ spectra recorded simultaneously in the LTQ ion-trap. Quantification was performed by stable isotope dilution on reconstructed ion-chromatograms generated in the Orbitrap.

### Microarray Analysis, RNA Labeling, and Isolation from BMDM Cultures

Incorporation of 4-thiouridine (Sigma) into newly-transcribed RNA including experiments of RNA time course microarray analysis of transcriptional changes in WT, Ifnar^−/−^, and Ifnb1^−/−^ BMDM following MCMV infection and quantitative RT-PCR using taqman primer probe sets (from Applied Biosystems) or for viral transcripts see Supplemental Experimental Procedures. Time course microarray analysis data are compliant with the National Centre for Biotechnology Information Gene Expression Omnibus (GEO) under SuperSeries accession number GSE42505 (SubSeries numbers GSE42503, GSE42504) (GEO, http://www.ncbi.nlm.nih.gov/geo/).

### Statistical Analysis of Transcription Factor Binding Site Overrepresentation

Entrez gene ID’s for 164 transcripts with a fold change of >2-fold in the Ifn-γ-treated BMDM samples between 0 and 30 min were imported into the oPOSSUM software tool (http://www.cisreg.ca/cgi-bin/oPOSSUM/opossum) and analyzed as described in Supplemental Experimental Procedures.

### Isolation and Quantification of Viral Genome Copy Number by Using qPCR

For DNA extraction, cells were extensively washed thrice 3 hr postinfection and total genomic DNA isolated and genome copy number normalized to input DNA or to gapdh using quantitative procedures described in Supplemental Experimental Procedures.

### Oxysterol Screening against MCMV, HSV-1, MHV-68, VZV, Influenza A, and Adenovirus

For these experiments, respective host cell systems were conducted in black 96-well plates using media conditions and oxysterol treatment as described in the figure legends and Supplemental Experimental Procedures. The IC_50_ values were calculated as the concentration at 50% viral inhibition. Cell viability and toxicity assays were determined using the CellTiter Blue (CTB, Promega) reagent.

### Metabolite Treatment of Cells

Murine embryo fibroblasts were infected with MCMV (MOI = 0.01). After adsorption, cells were washed 5 times with normal medium (DMEM, 10% FCS, L-Glutamine and Penicillin/Streptomycin). After washing, normal medium containing vehicle (Ethanol) or 25HC (1 μM) ± Geranylgeraniol (GGOH, 20 μM) (Sigma G3278) or Mevalonolactone (Mev, 20μM) (Sigma M4667), Farnesol (FOH, 20 μM) (Sigma F203) or Squalene (Sqle, 20 μM) (Sigma S3026) was added to the infected wells. After 4 days, supernatants were collected and MCMV titer calculated by plaque assay.

### Computational Prediction of Stat1 Binding Sites in the Ch25h Promoter

To analyze and predict potential Stat1 binding sites in the promoter of Human and Mouse *Ch25h* genes, we used the open source software Toucan. In brief, 1Kb 5′ *cis*-regulatory regions upstream of the transcriptional start sites of human (ENSG00000138135) or mouse (ENSMUSG00000050370) *Ch25h* were imported into Toucan. Predicted transcription factor binding sites were then identified in these sequences using the MotifLocator algorithm. Position Weight Matrices for this analysis were derived from the TransFac database V7.0 (public) and the background model used was either mouse or human DBTSS promoters depending on input sequence. A default stringency threshold of 0.9 was used for all predictions.

### Chromatin Immunoprecipitation (ChIP)

For ChIP of Stat1, 6 × 10^7^ Mϕ were used per ChIP, as detailed in the Supplemental Experimental Procedures, including the analysis of ChIP-Seq data for UCSC Browser image of Ch25h locus.

## Supplemental Information

Document S1. Figures S1–S4, Tables S1 and S2, and Supplemental Experimental Procedures

Movie S1. Visual Representation of Computational Modeling of Plaque Growth, Related to Figure 5
