# Supplementary material for: The Transcription Factor STAT-1 Couples Macrophage Synthesis of 25-Hydroxycholesterol to the Interferon Antiviral Response
Source: Immunity. 2013 Jan 24;38(1):106–18. doi: 10.1016/j.immuni.2012.11.004 (PMC3556782; doi:10.1016/j.immuni.2012.11.004)
Supplement: Document S1. Figures S1–S4, Tables S1 and S2, and Supplemental Experimental Procedures [file mmc1.pdf]

Immunity, Volume *38*

Supplemental Information

The Transcription Factor STAT-1 Couples Macrophage Synthesis of 25-Hydroxycholesterol to the Interferon Antiviral Response

Mathieu Blanc, Wei Yuan Hsieh, Kevin A. Robertson, Kai A. Kropp, Thorsten Forster, Guanghou Shui, Paul Lacaze, Steven Watterson, Samantha J. Griffiths, Nathanael J. Spann, Anna Meljon, Simon Talbot, Kathiresan Krishnan, Douglas F. Covey, Markus R. Wenk, Marie Craigon, Zsolts Ruzsics, Jürgen Haas, Ana Angulo, William J. Griffiths, Christopher K. Glass, Yuqin Wang, and Peter Ghazal

Supplemental Inventory

1. Supplemental Figures and Tables

Figure S1, Related to Figure 3

Figure S2, Related to Figure 4

Figure S3, Related to Figure 5

Figure S4, Related to Figure 6

Table S1, Related to Figure 6

Table S1, Related to Figure 6

2. Supplemental Experimental Procedures

3. Supplemental References

Figure S1.

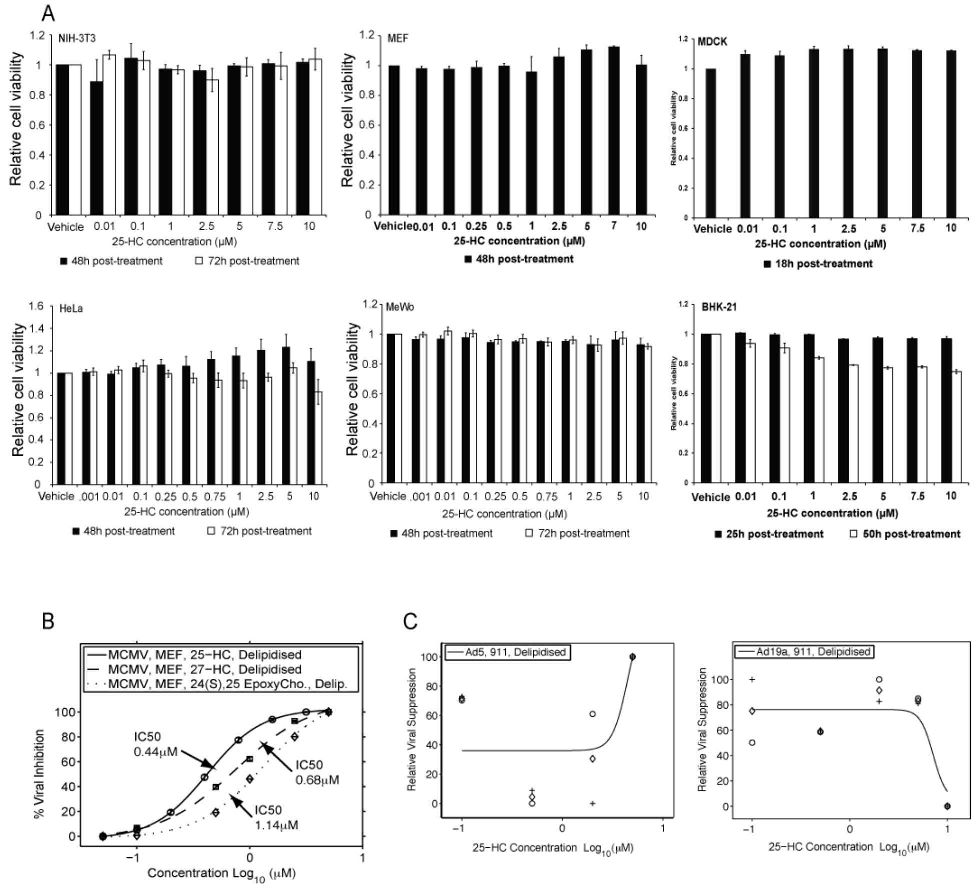

Figure S1, Related to Figure 3

(A) Analysis of 25-HC effects on cell viability. Cells were treated with 25-HC as indicated and viability analysed using the CellTiter Blue (CTB, Promega) assay as described in Experimental Procedures. Data are: NIH-3T3 mean +/- SEM for 2 experiments (8 replicates per experiment), MEF mean +/- SEM for 2 experiments (8 replicates per experiment), MDCK mean +/- SEM for 2 experiments (4 replicates per experiment), HeLa mean +/- SEM for 4 experiments (3 replicates per experiment), MeWo mean +/- SEM for 3 experiments (3 replicates per experiment) and BHK-21 mean +/- SEM for 2 experiments (8 replicates per experiment).

(B) Antiviral effects of SREBP targeting oxysterols in delipidised medium. Murine embryo fibroblasts were treated with 25-HC, 27-HC or 24(S), 25-epoxyCholesterol, infected with MCMV and viral growth was analysed as described in Experimental Procedures. Data are mean of 2 independent biological experiments and bars represent SEM (3 replicates per experiment).

(C) Adenovirus replication is unaffected by 25-HC treatment. 911 cells were infected with 10 physical particles/ cell of recombinant Human Adenovirus species C (Ad5-gfp) in 0.5ml DMEM + delipidised serum. Infected cultures were then harvested and virus titred as described in experimental procedures. Circles and crosses denote independent experiments and diamonds denote their mean (H) 911 cells were infected with 10 physical particles/ cell of recombinant Human Adenovirus species D (Ad19a-gfp) in 0.5ml DMEM + delipidised serum. Infected cultures were then harvested and virus titred as described in experimental procedures.

Figure S2

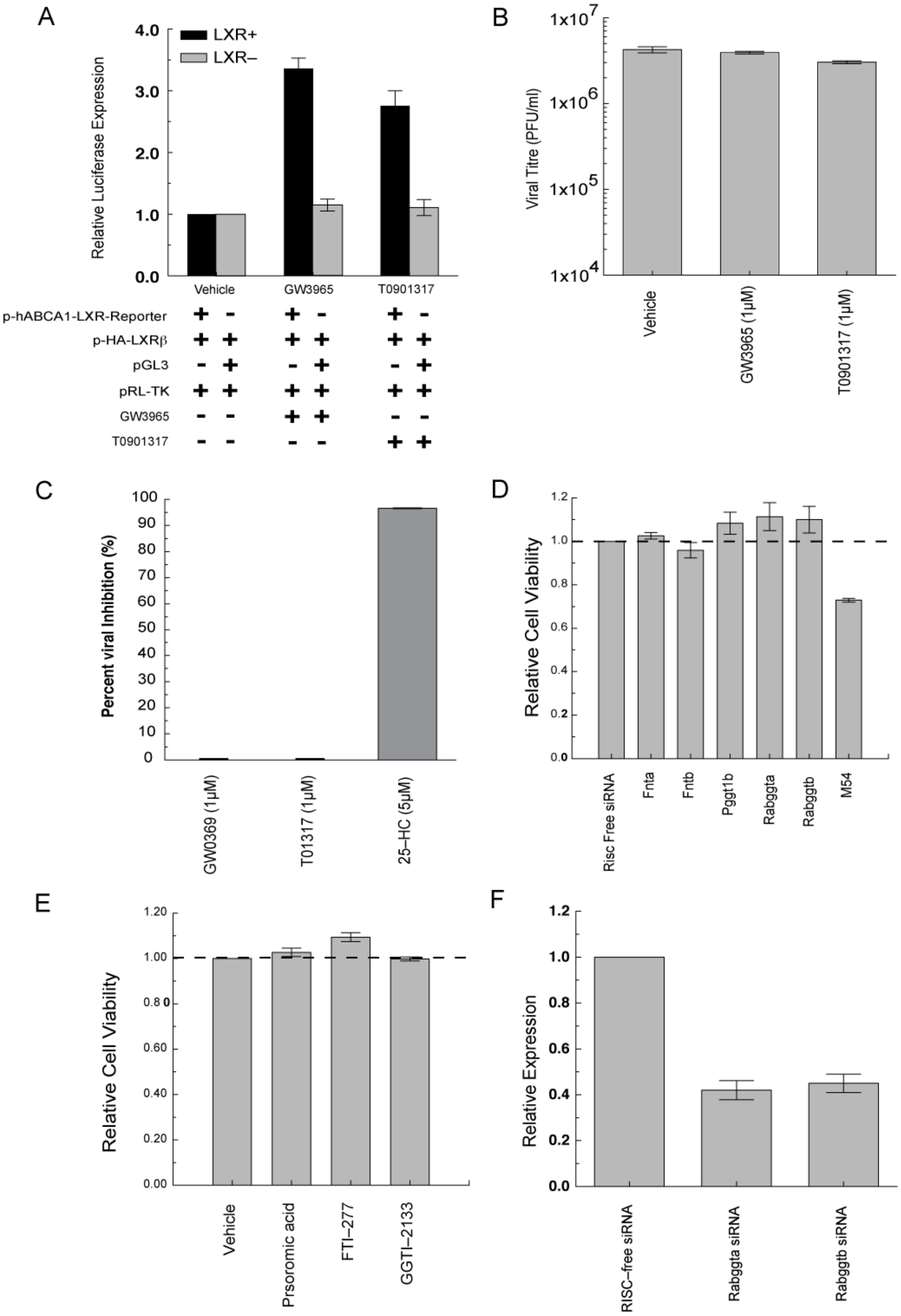
A
LXR+
LXR–
Relative Luciferase Expression
4.0
3.0
2.0
1.0
0.0
Vehicle
GW3965
T0901317
p-hABCA1-LXR-Reporter
p-HA-LXRβ
pGL3
pRL-TK
GW3965
T0901317
B
Viral Titre (PFU/ml)
1x10^7
1x10^6
1x10^5
1x10^4
Vehicle
GW3965 (1μM)
T0901317 (1μM)
C
Percent viral Inhibition (%)
100
90
80
70
60
50
40
30
20
10
0
GW0369 (1μM)
T01317 (1μM)
25–HC (5μM)
D
Relative Cell Viability
1.2
1.0
0.8
0.6
0.4
0.2
0.0
Risc Free siRNA
Fnta
Fntb
Pggt1b
Rabggta
Rabggtb
M54
E
Relative Cell Viability
1.20
1.00
0.80
0.60
0.40
0.20
0.00
Vehicle
Prsoromic acid
FTI–277
GGTI–2133
F
Relative Expression
1.2
1.0
0.8
0.6
0.4
0.2
0.0
RISC–free siRNA
Rabggta siRNA
Rabggtb siRNA

Figure S2. LXR Ligands Are Active but Do Not Inhibit MCMV Replication, Related to Figure 4

(A) Murine embryo fibroblasts were seeded in 96 well plates at $1x10^4$ cell/well. The next day, cells were transfected with 25ng of p-HA-LXRβ, 15ng of pRL-TK (Promega), 50ng of p-hABCA1-LXR-Reporter or pGL3 empty vector using jetPRIME transfection reagent (Polyplus, 114) according to manufacturer's protocol. 4 hours post transfection, transfection medium was replaced with fresh media. The next day, cells were stimulated with either vehicle or GW3965 (1μM) or T0901317 (1μM) for 18h. Cells were then lysed by added 60ul of 1X Passive Lysis Buffer (Promega, E1941) per well, followed by gentle shaking for 15 minutes. Firefly activity was assayed (30ul/well) using the Luciferase Assay System (Promega, E1501). For the transfection control, the Renilla activity (25ul/well) was assayed using coelenterazine in Experimental Procedures. Data are normalized to negative control wells treated with vehicle alone and are the mean of 3 independent experiments + SEM (6 replicates per experiment).

(B) Murine embryo fibroblasts were infected with MCMV (MOI = 0.1) as described in Experimental procedures. After adsorption, cells were washed 5 times with normal medium. After washing, cells were cultured in normal medium containing vehicle or GW3965 (1μM) or T0901317 (1μM) as indicated. 4dpi supernatants were collected and virus titre was quantitated by plaque assay as described in Experimental procedures. Data are the mean of 2 biological replicates +/- SEM.

(C) Murine embryo fibroblasts were pretreated with vehicle, GW3965 (1μM), T0901317 (1μM), or 25-HC (5μM) as indicated for 24h. Cells were then infected with Gluc-MCMV (MOI = 0.1) for 1h. After adsorption, cells were washed once with normal medium. After wash, 100ul of fresh medium containing GW3965 (1μM), T0901317 (1μM), or 25-HC (5μM) were added. Supernatant were collected at 24h.p.i. and GLuc activity was measured using the native form of the GLuc substrate coelenterazine as described in Experimental Procedures. Data are normalized to negative control wells treated with vehicle alone, with the vehicle set as 0% inhibition. Data are the mean of 2 independent experiments +/- SEM (8 replicates per experiment).

(D) RISC-Free, Fnta, Fntb, Pggt1b, Rabggta, Rabggtb, or MCMV M54 siRNA (25nM final concentration) were reverse-transfected into NIH/3T3 cells. 48h after transfection cell viability was assessed using a cell titre blue assay (Promega). Data are normalized to RISC-free treated wells and are mean of 6 independent experiments +/- SEM.

(E) MEF were pre-treated with 25-HC (5μM), or psoromic acid (20μM) as for 24h. Cell viability was then assessed using a cell titre blue assay (Promega). Data are normalized to vehicle treated wells and are mean of 2 independent experiments +/- SEM.

(F) RISC-Free, Rabggta or Rabggtb, (25nM final concentration) were reverse-transfected into NIH/3T3 cells. 48h after transfection the abundance of Rabggta or Rabggtb transcripts was quantitated by Q-RT-PCR. Data are normalized to RISC-free treated wells and are mean of 2 independent experiments +/- SEM.

Figure S3.

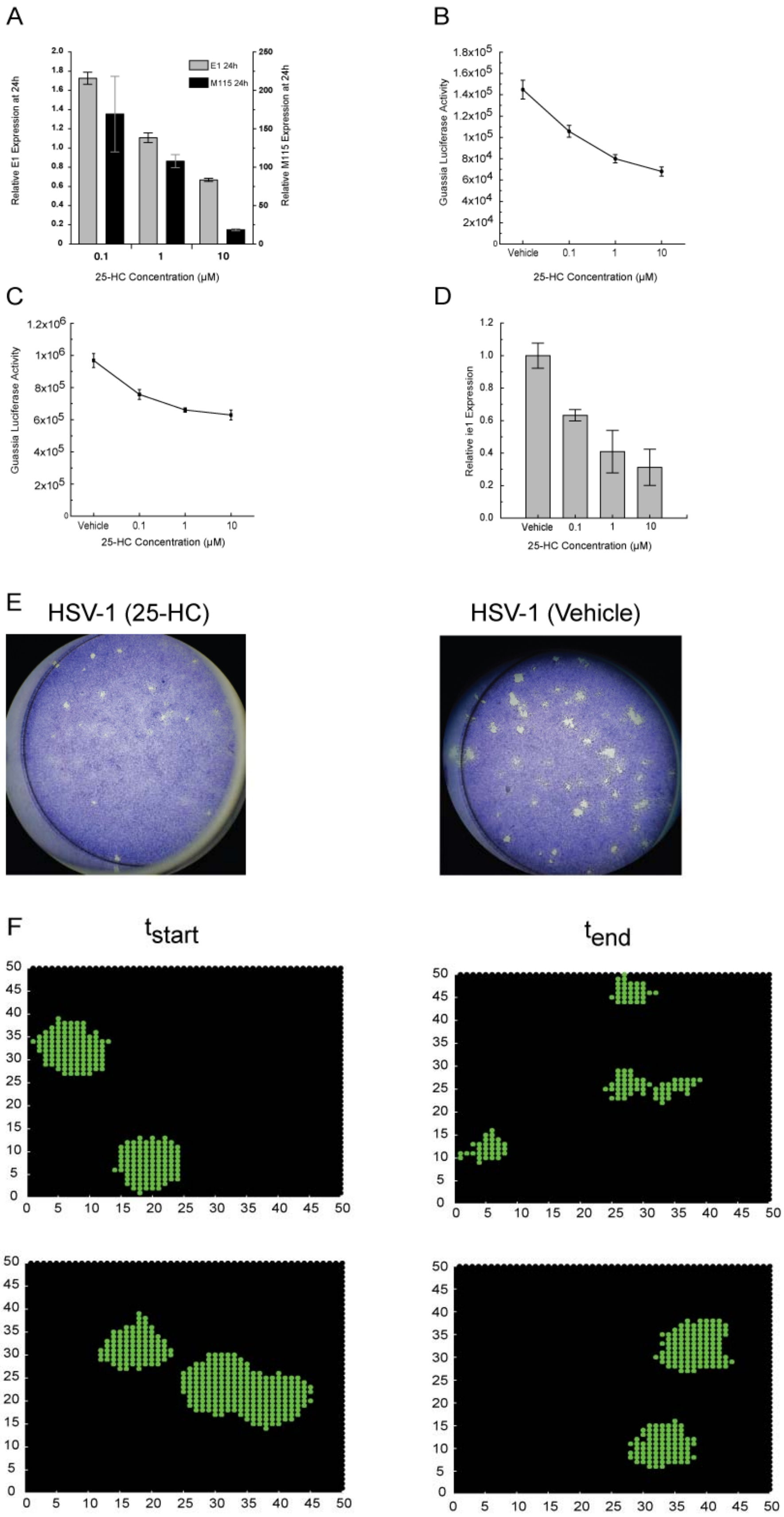
A
E1 24h
M115 24h
Relative E1 Expression at 24h
Relative M115 Expression at 24h
25-HC Concentration (μM)
B
Guassia Luciferase Activity
25-HC Concentration (μM)
C
Guassia Luciferase Activity
25-HC Concentration (μM)
D
Relative ie1 Expression
25-HC Concentration (μM)
E
HSV-1 (25-HC)
HSV-1 (Vehicle)
F
t_{start}
t_{end}

Figure S3. MCMV Gene Expression in NIH-3T3 Cells following Pretreatment with 25-HC, Related to Figure 5

(A) NIH-3T3 cells were pre-treated with the indicated doses of 25-HC for 24h, infected with MCMV (MOI = 1) and then cells were retreated with the same concentration of 25-HC as before - described in Experimental Procedures. After 24h, samples were harvested and abundance of MCMV E1 and M155 transcripts was analysed by Q-RT-PCR relative to the vehicle-treated MCMV infected cells. Graph shows fold changes in mRNA abundance (relative to vehicle control samples and duplexed with a GAPDH assay used as an internal normalisation control) of the E1 and M155 mCMV genes (n=3, error bars = mean +/-SE).

(B and C) Primary Murine embryo fibroblasts were cultured in 96 well plates for 24h then pre-treated with the indicated concentration of 25-HC for a further 24h. MEF were then infected with GLucMCMV (MOI = 0.1) washed 1x with normal medium and then re-cultured in normal medium with the same concentration of 25-HC as the pretreatment. After 2h (B) or 4h (C), the compete supernatant from each well was harvested and frozen. Guassia luciferase activity was then assayed as described in experimental procedures. Data are mean of 2 independent experiments +/- SE of mean (8 replicates per experiment).

(D) NIH-3T3 fibroblasts were cultured in 96 well plates for 24h then pre-treated with the indicated concentration of 25-HC for a further 24h. Cells were then infected with MCMV (MOI = 0.1) washed 1x with normal medium and then re-cultured in normal medium with the same concentration of 25-HC as the pretreatment. RNA was then harvested, isolated and IE1 transcript abundance quantitated as described in Experimental Procedures. Data are mean +/- SEM from a single run and are representative of multiple independent experiments.

(E and F) 25-HC treatment of cells reduces HSV-1 plaque size and number. A549 cells were treated with vehicle or 25-HC (5μM) for 24h. Cells were then infected with HSV-1-eGFP (C12) at MOI between 0.009 and 0.0003 and then cultured in medium containing vehicle or 25-HC (5μM) and 0.5% agarose. 72hpi, cells were fixed, stained and plaque numbers and diameters calculated for the MOI = 0.003 infection as described in Experimental Procedures. Representative photographic images of plaques from vehicle (E) or 25-HC (F) treated cells infected with an MOI = 0.003 are shown.

(G) Predictions by modelling of plaque size.

Top left - plaques formed in the absence of 25HC.

Top right - plaques formed in the presence of 5μM 25HC when cell permissivity and viral growth both contribute to the infectious transmission between cells.

Bottom left - plaques formed in the absence of 25HC.

Bottom right - plaques formed in the presence of 5uM 25HC when cell permissivity alone contributes to the infectious transmission between cells.

Figure S4.

A.

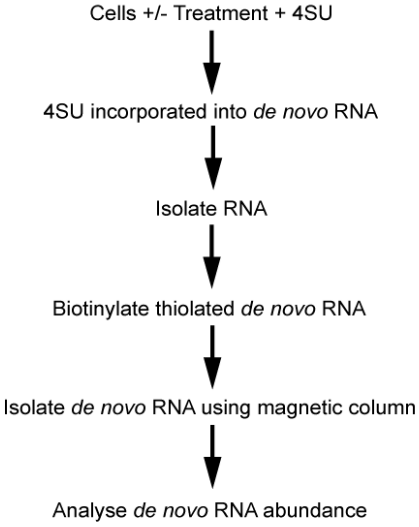

B. Ch25H Promoter: Predicted Stat1 binding Sites (+ strand) and primer locations

```
>M. musculus|chr19|34549416-34550616|Minus|Upstream:1000, Downstream:200
GGATCTTCTGTGCCGTGTATCCGCTACCCGGGGCTGATTTCTGTGCTCCACTCTCTAGGG

CACTCTCCTTGGATATAGTACTGACCTCCTGTGCTCAGTCCCTGGACGATCAGTCCCAGT

GTCTGAAAGGGTATCCAGGAGTTGAGGAGTAGGGAACAACTGAGGGTTGGTAAAAGTCGG

AGCCTGAGGGACCTCTAAGGAAATGGCAGGGGAGATGAGACATGTTCCGGGAGGCTATGA

GATTGCTCAGATCTTCTTCTCCACTGGCCTTTGCGGTTTGGCCCAATCGAGTGAGCCTTT

ACGGGTGTTAAGCCTCCGTTCTCCAACCCAGAGCAAAGGCTGGCAGTGCTGCCCTCTTGA

CCGCTGTCTTGCAGGATCTGATCTCTGCTCTTGACACCGCGGGCTTTAAGGGCACCAGAG

CGGGAGAGTCGTCCGCGCCAAAAGGCTGCGAGCCTGACTGCTCTTTTTTGGAGGATGCCA

GGTTCCCTCTCCTACAGGACTCAATCCCTGTCTGCAACGGACCCAGTACCAGCACTGTGG

CGTGAGGTACGCACACTCAGAGTCCCCCAAGGTGTAGAATAAGTCTTTAAACAGTCAAAA

ACAGATATTTTAAAACCCAGGGAATTCTGCTTTGGTTGAGAAACAGCGCCTTCTGAGAGC

GAAGAACTCATTAGTTTTTAGCGGTGATGGCGGGTGAACTGTCCCAAGGCAGAATCGCTC

TCTCCTGTGTCATTAGTACATCAGCTGAAAGGCAAGCAAACAGTTCTTTACGTCTGGGAA

AGGGCTTTAAGGGCCGCTGCTTGCGTGAAGGGGAGATTTGATGCAGCTATAAACCAGGAC

ATAGACAGGAACAAAATTCGGGTTATTTAAGCAAAATTCTTTAGCAGGGAAAGGGAGGTG

GGAAGGAGACGGAAGGGTGACGTCACTGGGGGGAGTGGCCACAGTCTTAAGAAAAGTCGG

CGGGGCTGGGGAGACCACAATTGTGGGACATAGTCTCAGCATGGGCTGCTACAACGGTTC

GGAGCTCCAAGACCTGGGCTGTTCCAGCCAGCTGCTCCTGCAGCCCCTCTGGGACACCAT

AAGGACAAGGGAGGCGTTCACGCGCTCACCCATCTTCCCAGTCACCTTTTCTATCATCAC

TTACGTGGGCTTCTGCCTACCGTTCGTGGTGCTGGACGTCCTGTATCCCTGGGTCCCCAT

C
```

C. PCR primers used for Q-RT-PCR detection of promoter sequences in ChIP DNA

Gene	Primer Sequence
Negative Control sense	GCAACTGTCATAGCTACCGTC
Negative Control anti-sense	GTGTATGAGGCTTTGGAAACT
Ch25h sense	GTGCCCTTAAAGCCCGCGGT
Ch25h anti-sense	GGCCTTTGCGGTTTGGCCCA
Nos2 sense	ACACGAGGCTGAGCTGACTT
Nos2 anti-sense	CACACATGGCATGGAATTTT
Tap1 sense	GGAAGTAGGCGTTATCTAGTGAGC
Tap1 anti-sense	GCCGAGTCCTCCCCTACT

D. Human Ch25H Promoter: Stat1 binding Site, Primers and amplicon (110bp)

```
>H. sapiens|chr10|90966871-90968071|Minus|Upstream:1000, Downstream:200
TAGCCTCCCGGGAGGCTTCAGGAACTCACATCTCTTTCCCTTGACGAACAACGCAGGTGG

TTTGCTGAATTAAGTGTTCCCGCCCCAAATTGAGATACAACGGGCTTTTTAGCCAGATTG

GCGCAGACTGGGAGTTAAATCCAGCATTTTCCCGGCAAAGCTGAGCCTGGCCGCCGCGGA

GCCCTCTTTCCGCTGTGCGGTGCCTCCGGCTCCGATCGCGGGCCCGGCGCCCCGGGACGC

GCCGCCGAGGGTAGCTACGCGGCCGGGCTGCGGCCTCTCCCGGAGAGCCTGCGTGCGTGG

TCTCTCTAGCTTGCTGCCCGCCGGCCAGGAAAACTCTGGGGACAAGCTGGAGCTGGCGGA

GGTTCCCATCCGGCCACCCTTCCCGTCTGATTTCCTGAGCTGACGGTCACCGCCGGCGTT

CGGGGACCAGCGGTTCCCTGTGCAAAGGGCTCGTTTCTCGATCGCCGCTGGATAGACCGC

AACCGGGTTCTCAGAAAACACCCGAGCTACAGACCGCATGTAGCTTTAAACAGTTTTTAA

AATGCGATTATGCTTGCGTCTTTATGAAAGCACATGGATGCAGTTATACGTATGCATTAA

AAAACACAAACAATTCTGCATTGATTGCAAAGGAGCGCTTTCTGGTGGCAATGTAAACGG

TCTAGTTTGAGACCCGTGATGACACACGTGAACTGAGTTGCCAAACTCATAGAAACTCCA

CACGGTAGATCTGTACATTGTGTCCCGTGTTAATTGTACGTCAACTGAAAAATCAGAACT

TACATGAAAAGTACGAAGTGCCACTGCATACACGCATATCTAAAAGCCGGTAAGATGCGG

CTATAGATGAGCACATTTACAGCAATGAAGGGCCACTCTGCCGGGTTGCTAGCTTATTCA

AGCTTCGCATCGCCGGGAAAGGGATGGAAAGGCAGCTGACATCACTCGGGGCGGGGCCGT

GAGTCCTGTGAAAAGTCGATGGGACTCGAGCAGCAGCCTCAGCTGCCTTGCACAGCCTCG

CAATGAGCTGCCACAACTGCTCCGACCCCCAGGTCCTTTGCAGCTCCGGGCAGCTGTTCC

TGCAGCCCCTCTGGGACCACCTGAGGAGCTGGGAGGCCCTCCTACAGTCGCCCTTCTTCC

CGGTCATCTTCTCCATCACCACATACGTGGGCTTTTGCCTGCCCTTCGTGGTCCTGGATAT
```

Figure S4, Related to Figure 6

(A) Workflow for labelling and isolation of newly transcribed (*de novo)* RNA.

(B) Analysis of the murine Ch25h promoter. A 1Kb region of the Ch25h promoter (upstream of the transcriptional start site - arrow) was input into Toucan open-source software and putative Stat1 binding sites identified described in Experimental Procedures. Boxes indicate predicted Stat1 binding sites (for positive strand) and lines indicate position of primers used for quantitative analysis of Ch25h DNA.

(C) Primers used for the analysis of DNA from Chromatin Immuno-precipitation experiments.

(D) Analysis of the Human Ch25h promoter. A 1Kb region of the *Ch25h* promoter (upstream of the transcriptional start site - arrow) was input into Toucan open-source software and putative Stat1 binding sites identified described in Experimental Procedures. Boxes indicate predicted Stat1 binding sites (for positive strand).

Table S1. Fifty-one Transcripts with Similar Synthesis Profiles to *Ch25h* following an 8 hr IFNγ Treatment of Bone Marrow-Derived Macrophages, Related to Figure 6

Gene Symbol	Entrez Gene ID		Gene Symbol	Entrez Gene ID
Apobec3	80287		*Itga4*	16401
Arid5a	214855		*Map3k8*	26410
BC013712	230787		*Mlkl*	74568
Casp1	12362		*Mt2*	17750
Casp4	12363		*Nampt*	59027
Ccdc25	67179		*Niacr1*	80885
Ccl12	20293		*Nlrc5*	434341
Cdc42ep2	104252		*Obfc2a*	109019
Ch25h	12642		*Olfr56 /// Ifi47*	15953
Cish	12700		*Pml*	18854
Cxcl10	15945		*Ralgds*	19730
F3	14066		*Samhd1*	56045
Fam46c	74645		*Slc28a2*	269346
G530011O06Rik	654820		*Sod2*	20656
Gdap10	14546		*Sp110*	109032
Gja1	14609		*Stx11*	74732
Gm11428	100034251		*Tagap /// Tagap1*	380608
Gm12250	631323		*Tap1*	21354
Gm5431	432555		*Tmem173*	72512
Gm8995	668139		*Tmem2*	83921
Icam1	15894		*Tnfaip2*	21928
Il10ra	16154			
Il15	16168			
Il1a	16175			
Irf1	16362			
Irf8	15900			
Irgm1	15944			

A microarray analysis of *de novo* transcription during the first 8 hours after Ifnγ stimulation of macrophages followed by a partitioning around median (PAM) clustering analysis of temporal differential synthesis profiles identified 51 transcripts kinetically related to the hydroxylase gene, *Ch25h.*

Table S2. One hundred and nine Transcripts Increased by >2-fold during the First 30 Minutes after Ifnγ Treatment and Used by oPOSSUM for the Statistical Analysis of Transcription Factor Binding Site Overrepresentation, Related to Figure 6

Gene symbol	Entrez Gene ID		Gene symbol	Entrez Gene ID		Gene symbol	Entrez Gene ID
2610507B11Rik	72503		*Hspa14*	50497		*Tap1*	21354
9030418K01Rik	71532		*Hspa2*	15512		*Tfrc*	22042
Adora3	11542		*I830077J02Rik*	433638		*Tinf2*	28113
Ahr	11622		*Icam1*	15894		*Tmbim6*	110213
Apobec3	80287		*Il1a*	16175		*Tmem173*	72512
Arid5a	214855		*Il1rn*	16181		*Tmem88*	67020
Aste1	66595		*Il4ra*	16190		*Tnfrsf1a*	21937
Atrip	235610		*Irf1*	16362		*Trim21*	20821
B4galt3	57370		*Irf8*	15900		*Ugt1a10*	394430
Batf2	74481		*Irg1*	16365		*Zbp1*	58203
BC013712	230787		*Kdm6b*	216850		*Zfp281*	226442
Birc3	11796		*Lcp2*	16822			
Casp4	12363		*Lsmd1*	78304			
Ccdc25	67179		*Map3k8*	26410			
Cd40	21939		*Mcts2*	66405			
Cd69	12515		*Mmp13*	17386			
Cd80	12519		*Mob3c*	100465			
Ch25h	12642		*Mt2*	17750			
Cish	12700		*Myd88*	17874			
Clec4n	56620		*Nfil3*	18030			
Crem	12916		*Niacr1*	80885			
Csrnp1	215418		*Noc4l*	100608			
Cxcl10	15945		*Nop58*	55989			
Cxcl9	17329		*Obfc2a*	109019			
Daxx	13163		*Osgin1*	71839			
Dcun1d1	114893		*Paox*	212503			
Dek	110052		*Parp10*	671535			
Dok2	13449		*Parp9*	80285			
Dr1	13486		*Pla2g15*	192654			
Dtx3l	209200		*Plec*	18810			
Ednrb	13618		*Pml*	18854			
Egr2	13654		*Ppp1r3d*	228966			
Eif2ak2	19106		*Prpf31*	68988			
Emb	13723		*Ralgds*	19730			
Fam54a	71804		*Rap2c*	72065			
Farsa	66590		*Rhoh*	74734			
Fbxl5	242960		*Ripk1*	19766			
Fgl2	14190		*Rnasel*	24014			
Fscn3	56223		*Rnf31*	268749			
Furin	18550		*Samhd1*	56045			
Fzd7	14369		*Sav1*	64010			
Gbp5	229898		*Sbno2*	216161			
Gcc1	74375		*Slc26a2*	13521			
Gosr2	56494		*Slc31a1*	20529			
Gpr146	80290		*Slfn5*	327978			
H13	14950		*Socs1*	12703			
Hbegf	15200		*Socs3*	12702			
Heatr1	217995		*Sod2*	20656			
Hprt	15452		*Stk19*	54402			

Supplemental Experimental Procedures

Cell Propagation and Culture

BMDM were derived from femur and tibia isolated from C57BL/6 mice. Bone marrow cells were flushed with DMEM supplemented with 1% PS using a 5-ml syringe and a 25 gauge needle. Cells were centrifuged for 5 minutes and the supernatant was discarded. The cell pellet was then resuspended in DMEM/F12 + GlutaMAX (Lonza, Vervier, Belgium) supplemented with 10% Fetal Bovine Serum (FCS), 10% L929 conditioned medium (containing Colony-stimulating factor Csf1) and Penicillin/Streptomycin. NIH-3T3 fibrobasts were obtained from LGC Standards (Teddington, UK) and cultured in DMEM (Lonza, Vervier, Belgium) supplemented with 10% Bovine Serum (Lonza), 2mM L-Glutamine and Penicillin/Streptomycin. HeLa cells were cultured in DMEM supplemented with 5% FCS, 2mM L-Glutamine and Penicillin/Streptomycin. BHK-21 cells were cultured in EMEM (Lonza, Vervier, Belgium) supplemented with 10% FCS, 2mM L-Glutamine and Penicillin/Streptomycin. MeWo cells were cultured in EMEM supplemented with 10% FCS, 2mM L-Glutamine and Penicillin/Streptomycin. Mouse embryo fibroblasts (MEFs) derived from the embryos of timed pregnant C57BL/6 mice on day 14-17 of gestation, were cultured in Eagle's minimum essential medium (EMEM) supplemented with 10% FCS, 50U/ml penicillin and streptomycin and 2mM glutamine. MEFs were used at passage 3 post-isolation for experiments. MDCK cells are grown in DMEM, 10% FCS, 2mM L-Glutamine and Penicillin/Streptomycin. RAW264.7 cells were cultured in DMEM supplemented with 10% FCS, L-Glutamine and Penicillin/Streptomycin.

Cytokines, Drugs, Oxysterol and Liver-x Receptor Ligand Treatments

Ly295427 and Ly306039 were a kind gift from David Russell. 25-Hydroxycholesterol (25-HC) was purchased from Avanti Polar Lipids. Ly295427, Ly306039 and 25-HC were resuspended in ethanol to a stock concentration of 2.5mM, stored under Argon and used within a month. The *ent*-25-HC was prepared as described previously except that one of the terminal methyl groups in the side chain was trideuterated (Westover and Covey, 2006). GW3965 (Sigma-Aldrich, UK) was resuspended in DMSO (10mM stock) and T0901317 (Tocris Bioscience, Bristol, UK) was resuspended in 100% ethanol (5mM stock). Both GW3965 and T0901317 were diluted in medium prior to use. Psoromic acid (Santa Cruz Biotech, USA), FTI-227 (MerckMilipore, UK) and GGTO-2133 (Sigma, UK) were dissolved in DMSO. Murine recombinant Interferon gamma (IFNγ) (Perbio Science) and Interferon Beta 1 (IFNβ) (Stratech, UK) were diluted in complete medium and were added to cells at a final concentration of 10 U/ml or 25 U/ml respectively.

Reporter Viruses and Viral Plaque Assay

The construction of the GFP-encoding Murine Cytomegalovirus (MCMV-GFP, originally named: pSM3fr-rev) used in this study was previously described (Angulo et al., 2000). The virus was propagated in mouse NIH-3T3 fibrobasts. The construction of GFP-encoding Murine Gammaherpesvirus 68 (MHV-68-GFP, originally named: LHΔgfp) used in this study was previously described (Dutia et al., 2004). The virus was propagated in baby hamster kidney cells (BHK-21). The Gaussia luciferase (GLuc) reporter virus (GLuc-MCMV) was generated by site-specific homologous recombination as previously described and was obtained from M. Messerle (Borst et al., 2007; Kropp et al., 2011). The amount of infectious MCMV/MHV-68 present in the viral stock was quantified by plaque assay on p53 -/- MEF monolayers in 48-well plates. VZV-GFP (vaccine strain Oka (Zerboni et al., 2000)) was propagated and titred in the human melanoma-derived MeWo cells. HSV-1-eGFP (C12) was

propagated and titred in Vero cells (Arthur et al., 2001). A/WSN/33 (H1N1) influenza virus was obtained from Dr. B. Dutia, Roslin Institute, University of Edinburgh and was propagated and titred in MDCK cells.

Plaque Reduction Assay

MCMV **-** p53 -/- murine embryo fibroblasts were seeded in 6 well plates. After 24h cells were infected with MCMV-GFP (MOI = 0.001) and overlayed with medium containing 2.5% agarose and 0.1, 1, 2.5 or 5µM 25-HC. Foci of infection were counted 24hpi using a fluorescence microscope, a Zeiss Axio Observer Z1 inverted microscope (Carl Zeiss, Germany). Microscope control and image capture were undertaken using Axiovision Software (Carl Zeiss, Germany). All images were treated in an identical fashion and overlays were produced in Photoshop CS3 (Adobe, USA)..

Plaque diameter was measured 3 days post infection. Thirty images per concentration of 25-HC were captured and diameters were quantified using ImageJ software.

HSV-1 - A549 cells were seeded at 1 x 10^5 cells/well in 24-well and cells infected with HSV-1 C12 at MOI between 0.09 and 0.0027, and replaced with growth medium containing 0.5% agarose and either ethanol (vehicle) at 0.05%, or 5 µM 25OH. Cells were fixed 72h post-infection in 1% formaldehyde for 1h before agarose plugs were removed and plaques analysed by fluorescent microscopy. Plaque numbers and dimensions were measured using ImageJ software.

GLuc Reporter Assay

GLuc activity was measured using the native form of the GLuc substrate coelenterazine (C-7001; Biosynth, Staad, Switzerland) as described previously (Kropp et al., 2011).

Measurement of Oxysterols

Extraction of Oxysterols and Sterols

Following treatments, cells (10^6) were separated from medium (retained for subsequent extraction) and washed twice with ice cold PBS (3ml). A further 1.6ml of ice cold PBS was added to each plate and cells scraped into a 2ml microcentrifuge tube. The cells were centrifuged at 600g (4° C) for 5 min, the supernatant discarded, and 1ml of 99.9% ethanol added to the cell sediment. To the cell sediment in 1ml of 99.9% ethanol, 1.1ml of 99.9% ethanol containing 20ng of $[^2H_6]$24(R/S)-hydroxycholesterol and 1µg of $[^2H_7]$cholesterol (both Avanti Polar Lipids) was added, with sonication in an ultrasonic bath (5 min). Following centrifugation at 14,000g (4° C) for 60 min, 0.9ml of water was added drop-wise to the supernatant to give a solution of 3ml of 70% ethanol.

To extract secreted oxysterols, incubation medium (2ml) was added drop-wise, with sonication in an ultrasonic bath (5 min), to 4.7ml of 99.9% ethanol containing 20 ng of $[^2H_6]$24(R/S)-hydroxycholesterol and 1µg of $[^2H_7]$cholesterol to give a solution of 6.7ml of 70% ethanol.

Separation of Oxysterols from Sterols

The cell extract in 3ml of 70% ethanol was applied to a previously washed (4ml 99% ethanol) and conditioned (6ml 70% ethanol) Certified Sep-Pack C_{18} cartridge (Waters). The flow-through and a column-wash of 4ml of 70% ethanol were collected and combined. This constitutes the oxysterol fraction (7ml 70% ethanol). Following a further column wash with a

second 4ml of 70% ethanol, cholesterol and sterols of similar polarity were eluted with 2ml of 99.9% ethanol. Both fractions were dried under reduced pressure.

Secreted sterols and oxysterols in 6.7ml of 70% ethanol were fractionated in an identical fashion to those derived from cells except the oxysterol fraction consisted of the 6.7ml 70% ethanol column flow-through and 0.3ml 70% ethanol column-wash.

Dried oxysterol and sterol fractions were then derivatised with Girard P reagent and analysed by LC-MS(MS^n) on a LTQ-Orbitrap Velos as previously described (Ogundare et al., 2010). Mass spectra were recorded at high resolution in the Orbitrap analyser, and MS^n spectra recorded simultaneously in the LTQ ion-trap. Quantification was performed by stable isotope dilution on reconstructed ion-chromatograms generated in the Orbitrap.

Time-Course Microarray Analysis of Transcriptional Changes in Wild Type, Ifnar-/- and Ifnb1-/- BMDM following MCMV Infection

Wild-type, Ifnar-/- and Ifnb1-/- macrophages were derived from mice as described and grown in 24 well plates. After 7 days of culture, BMDM were infected or mock infected with MCMV-GFP (MOI = 1). Cells were then harvested at 2, 4, 6, 8, 10 and 24 hours post-infection for the isolation of RNA and microarray analysis . In brief, arrays were normalised using the gcRMA algorithm (Zhijin Wu et al., 2004) and imported into Partek Genomics Suite (Partek, USA) for downstream analysis with. the analysis of statistical pathway over-representation in Ingenuity Pathway Analysis Software (Huang da et al., 2009).

RNA Labelling and Isolation from IFN-Gamma Treated BMDM

Incorporation of 4-thiouridine (Sigma) into newly-transcribed RNA was undertaken as described by Dölken *et al*. (Dolken et al., 2008). RNA labelling in BMDM during 30 minute intervals of the time course was undertaken by addition of 200µM 4-Thiouridine.

Newly Transcribed RNA Labelling for Microarray Analysis

The objective of this study was to capture dynamic changes over time in treated versus untreated samples. Since conventional clustering algorithms do not typically assign significance to differential expression of two conditions over time (on an individual gene basis) the MaSigPro algorithm was used (Conesa et al., 2006). This algorithm allowed the measurement of a difference between treated and untreated samples in relation to the effects of time and provided a list of genes with p values for use in subsequent analyses. In brief, a 4th-order polynomial model was fitted to the *de novo* RNA time course data from the 12,472 gene probes identified above. Significant differences for each gene between IFNγ and control time courses were then identified through a step-wise 2-ways backward goodness-of-fit regression at $p<=0.05$ and $r^2>=0.9$. The output of this analysis was 2086 probes with notable differential profiles of expression across the 8 hours of IFNγ stimulation. A non-parametric partitioning around median (PAM) clustering algorithm was then applied in R (using the package 'cluster') to partition the 2086 relative transcript gene expression profile changes (across time) into distinct patterns of co-expressed genes, allowing the identification of notable expression patterns.

Statistical Analysis of Transcription Factor Binding Site Overrepresentation

Entrez gene ID's for 164 transcripts with a fold change of >2 fold in the IFNγ treated BMDM samples between 0 and 30 minutes were imported into the oPOSSUM software tool (http://www.cisreg.ca/cgi-bin/oPOSSUM/opossum). In brief, oPOSSUM is a software tool

for identifying statistically over-represented transcription factor binding motifs in lists of imported genes. It does this by implementing statistical tests comparing the imported list with a background dataset compiled from all one-to-one human to mouse orthologs in Ensembl. In this study oPOSSUM recognised 109/164 imported genes for further processing. An analysis was then undertaken using default parameters and selecting for a stringent combined output z score of >=10 and a Fishers score of <0.01 (Ho Sui et al., 2007).

Quantitative RT-PCR

Taqman Primer probe sets were purchased from Applied Biosystems, Warrington, UK (Mouse Assay ID: *Ch25h*: Mm00515486_s1, *Hmgcr*: Mm01282499_m1, *Actb*: Mm00607939_s1). For each sample, QRT-PCR was performed in 20µl volumes using 96-well Non-Skirted, White PCR Plates (ABgene, UK) and MicroAmp Optical Caps (Applied Biosystems, UK). For 1 reaction, 1µl of diluted total RNA samples (50 ng) was added to 10µl of Brilliant III QRT-PCR 1-step master-mix, 1µl of a Taqman primer/probe set (Applied Biosystems, UK), 1µl RT/RNase Block Enzyme Mixture (Agilent, UK) and 7µl RNase-free H_2O. Reverse transcription was undertaken for 30 minutes at 50° C after which an incubation at 95° C for 3 minutes was used to activate the RNA polymerase. Samples were then subject to 45 cycles under Taqman standard conditions (combined annealing and primer extension phase at 60° C for 20 seconds and a short denaturation at 95° C for 20 seconds). Stratagene MXPro software was used to analyse the data. Threshold determinations and differences in transcript abundance relative to β-actin (*Actb*) were automatically performed by software for each reaction. IE1 gene expression levels were analyzed by relative quantitative real-time PCR using TaqMan primers and probe combinations as described elsewhere (Kropp et al., 2009).

Isolation and Quantification of Viral Genome Copy Number using qPCR

Cellular DNA was extracted with the Qiagen QIAamp DNA extraction kit (Cat N. 51304) by using the QIAcube running with the QIAamp blood and body fluid protocol (2009; available on Qiagen website) following manufacturer's instructions. A custom qPCR assay was developed to measure the quantity of MCMV genomes in infected cell samples. Briefly, a reference DNA plasmid containing the MCMVm115 (gL) gene was constructed. The concentration of purified DNA was determined using NanoDrop spectrophotometer (NanoDrop Technologies, DE, USA). The plasmid size (4167nt) and the DNA concentration were used together to calculate DNA copy number per µl in the purified plasmid stock. To perform qPCR, the purified MCMV M115 plasmid was first linearized by restriction digestion, followed by serial dilutions in RNase-free water (10^{-1} to 10^{-8}). The 82bp fragment from the M115 (gL) gene was amplified using a custom Taqman gene expression assay (Applied BioSystems, CA, USA) with primer sequences 5' GAGCTCAACGACGAGTTCCT 3' (forward) and 5' GCATCAGCGTCAGCAGAAC 3' (reverse). The qPCR data from the MCMV M115 reference plasmid dilution series was used to generate a standard curve with cycle threshold (Ct) values plotted on the y-axis against MCMV M155 copy number on the x-axis. At this stage Ct values derived from any sample could be inserted into the formula (as Y) to calculate copy number per µl compared to the reference plasmid. To control for variation in input material, data were normalised either to amount of input DNA or to abundance of GAPDH (representing host DNA).

Oxysterol screening against MCMV, HSV-1, MHV-68, VZV, Influenza A and Adenovirus.

NIH-3T3 fibroblasts were seeded in black 96-well plates at $3x10^4$cells/well for 24h. After the removal of media, cells were infected with GFP tagged murine cytomegalovirus (MCMV-GFP) [multiplicity of infection (MOI = 0.05] for 1h at 37° C. Cells were then incubated with DMEM [supplemented with 3% delipidised FCS (S5394, Sigma), L-Glutamine and Penicillin/Streptomycin] with appropriate concentrations of oxysterols.

For the HSV-1 experiment, HeLa cells were seeded in black 96-well plates at $2x10^4$ cells/well for 24h. For pre-treatment, cells were incubated for 24h with 25-HC diluted in DMEM/F-12 (supplemented with 3% delipidised FCS, 15mM HEPES, L-glutamine and Penicillin/Streptomycin). Cells then infected with HSV-1-eGFP diluted to MOI 2 for 1h before media was removed and replaced with the appropriate concentrations of 25-HC diluted in medium supplemented with delipidised serum. For non-pre-treatment, cells were infected as above and media replaced with the appropriate concentrations of 25-HC diluted in medium supplemented with normal FCS.

For the MHV-68 experiment, BHK-21 cells were seeded in black 96-well plates at $3x10^4$cells/well for 24h. After the removal of media, cells were infected with MHV-68-GFP (MOI = 0.05) 1h at 37° C. Cells were then incubated with EMEM (supplemented with 3% delipidised FCS, L-Glutamine and Penicillin/Streptomycin) with appropriate concentrations of 25-HC.

For the VZV experiment, MeWo cells were seeded in black 96-well plates at $2x10^4$ cells/well for 24h. For pre-treatment, cells were incubated for 24h with 25-HC diluted in EMEM (supplemented with 3% delipidised FCS, 1% non-essential amino acids, L-glutamine and Penicillin/Streptomycin). Cells were then infected with 130 colony forming units of VZV-eGFP-infected MeWo cells diluted in EMEM containing the appropriate concentrations of 25-HC diluted in EMEM (supplemented with 3% delipidised FCS, 1% non-essential amino acids, L-glutamine and Penicillin/Streptomycin). For non-pre-treatment, cells were infected as above and media was replaced with the appropriate concentrations of 25-HC diluted in medium supplemented with normal FCS.

Virus replication was monitored as a function of eGFP fluorescence hours post-infection (24-72h for MCMV, 19-48h for MHV-68, 20-60h for HSV-1 and 20-60h for VZV) using the POLARstar OPTIMA plate reader (BMG Labtech). Virus replication slopes over the linear phase were calculated and normalised to vehicle treated cells, and the mean replication slope from three independent experiments was calculated.

For the Influenza experiment, MDCK cells were seeded in 6 well plates at $5x10^6$cells/well and infected with Influenza A/WSN/33 (H1N1) at MOI = 0.01 in serum-free DMEM and incubated with serum-free DMEM containing 12.5mM HEPES, 2.5ng/ml N-acetyltrypsin, and appropriate concentrations of 25-HC. Supernatant was collected at 16hpi for subsequent plaque assay on fresh MDCK cells.

To analyze Adenovirus (Ad) replication in presence of 25-HC, 911 cells were plated onto 24 well plates at density of approximately 50,000 cells/well in DMEM containing 10% FCS (Fallaux et al., 1996). 12h after plating, cells were washed and infected with 10 physical particle/cell recombinant human Ads representing species C (Ad5-gfp) and D (Ad19a-gfp) diluted in 0.5 ml DMEM containing 5% delipidized serum (Sigma, Germany) and 0.01, 0.1, 0.5, 2, 5 or10μM 25-HC. Infected cultures were harvested 12, 24, 48, 72h after infection and their infectivity determined by standard TCID50 assay using 293 cells. Ad5-gfp was purchased from Sirion Biotech (Germany) and Ad19a-gfp was propagated as described previously (Ruzsics et al., 2006).

Viral inhibition dose curves were calculated from biological and technical replicate data. Sigmoidal functions of the form y=a/(b+exp(-c(x-d)))+e were used throughout and curves fitted using the MATLAB curve fitting toolbox (MathWorks, UK). For biological replicates, data was divided in each dose-response set by the control value to give a data set with values in the interval [0,1]. Each biological replicate had been obtained with a different set of metabolite concentrations. To facilitate comparison, we fitted a sigmoidal curve to each and sampled from these curves at intervals of half of an order of magnitude, calculating the mean and standard error. The resulting data points, curve and errors were then rescaled so that the viral inhibition extended from 0% to 100%.

For technical replicates, the viral inhibition values were averaged across the replicates and average values were scaled to span the interval [0%, 100%]. The standard error was calculated from the replicates and also rescaled. IC50 concentrations values were calculated as the point of 50% viral inhibition.

Metabolite Treatment of Cells

MEFs were infected with MCMV (MOI=0.01) and, after washing, normal medium containing vehicle (Ethanol) or 25-HC (1μM) +/- Geranylgeraniol (GGOH - 20μM) (Sigma G3278) or Mevalonolactone (Mev - 20μM) (Sigma M4667), Farnesol (FOH - 20μM) (Sigma F203) or Squalene (Sqle - 20μM) (Sigma S3026) was added to the infected wells. After 4 days, supernatants were collected and MCMV titre calculated by plaque assay as described in Experimental Procedures.

Cell Viability and Cell Death Assay

Cell viability was determined using the CellTiter Blue (CTB, Promega) reagent, and fluorescence was measured in a POLARstar OPTIMA plate reader. Readings were normalised to viability of vehicle treated cells, and mean cell viability was calculated over three replicates. Cell death was assessed using the Cell Death Detection ELISA PLUS kit (Roche, 11774425001) according to manufacturer's protocol. As per manufacturer's instructions, mean viability <70% was considered cytotoxic.

Computational Prediction of Stat1 Binding Sites in the Ch25h Promoter

To analyse and predict potential Stat1 binding sites in the promoter of Human and Mouse *Ch25h* genes, the open source software Toucan was used (Aerts et al., 2005). In brief, 1Kb 5' Cis-regulatory regions upstream of the transcriptional start sites of Human or Mouse *Ch25h* were imported into Toucan. Predicted transcription factor binding sites were then identified in these sequences using the MotifLocator algorithm. Position Weight Matrices for this analysis were derived from the TransFac database V7.0 (public) and the background model used was either Mouse or Human DBTSS promoters (0) depending on input sequence. A default stringency threshold of 0.9 was used for all predictions.

Chromatin Immunoprecipitation (ChIP)

For ChIP of Stat1, $6x10^7$ macrophages were used per ChIP, as described previously (Robertson et al., Nature Methods 2007). For preparation of Dynabead (Invitrogen)-antibody complex, Dynabeads Protein G and 3μg of each Stat1 antibody (catalog # sc-345 from Santa Cruz and catalog # 9172 from Cell Signaling) were incubated in 0.5% BSA/PBS for 1h at 4°C on rotator, then washed twice with 0.5% BSA/PBS and brought up to the original volume with 0.1% BSA/PBS. The beads were collected by magnet, the supernatant

discarded, and resuspended in 500 µl RIPA buffer (10mM Tris-Cl, pH 8.0, 140mM NaCl, 1% Triton X-100, 0.1% SDS, 1% deoxycholic acid, 0.5mM PMSF, 1mM DTT, 0.1mM sodium orthovanadate and protease inhibitors). The beads were washed six times with RIPA buffer (10 mM Tris-Cl, pH 8.0, 140mM NaCl, 1% Triton X-100, 0.1% SDS, 1% deoxycholic acid, 0.5mM PMSF, 1mM DTT, 0.1mM sodium orthovanadate and protease inhibitors), and twice each with TE plus 0.2% triton X-100 and TE plus 50mM NaCl. Immunoprecipitated chromatin was eluted twice with 100 µl elution buffer each (TE, 2% SDS) into fresh tubes for 30min and 10min, respectively, eluates were pooled, the Na^+ concentration was adjusted to 300mM with 5M NaCl and crosslinks were reversed overnight at 65° C in a hybridization oven. The samples were sequentially incubated at 37° C for 1h each with 0.33mg/ml RNase A and 0.5mg/ml proteinase K. The DNA was isolated using the ChIP DNA Clean & Concentrator (Zymo Research) according to the manufacturer's instructions. Negative control primer sequences alongside sequences for the Q-RT-PCR detection of *Ch25h*, *Nos2*, *Tap1* are presented in Supplementary Figure S6.

Analysis of ChIP-Seq Data for UCSC Browser Image of Ch25h Locus

ChIP-Seq data for H3K4me2-MNase ChIP-Seq (Kaikkonen et al, Epigenetic enhancer mark deposition dependent on eRNA elongation (unpublished in preparation)), H4K5Ac-ChIP-Seq (Spann et al, unpublished in preparation), and H3K4me3-ChIP-Seq (Escoubet-Lozach et al, PLoS Genetics 2011) provided from unpublished or published manuscripts as indicated. Analysis was performed using HOMER and the detailed instructions for analysis can be found at http://biowhat.ucsd.edu/homer/ (Heinz et al, Molecular Cell 2010). Each sequencing experiment was normalized to a total of 10^7 uniquely mapped tags by adjusting the number of tags at each position in the genome to the correct fractional amount given the total tags mapped. Sequencing experiments were visualized by preparing custom tracks for the UCSC Genome browser.

Computational Modelling of Virus spread.

Building the model

We built a 2D square lattice of cells and assigned to each cell a parameter, I, which held a value between 0 and 1, where 0 describes the absence of infection and 1 describes full infection. Each cell was subject to infection from its four nearest neighbors and so in a time step, dt, for a cell located at the coordinates (i, j), I_{ij} was updated according to

$$I_{i,j}(t+dt) = I_{i,j}(t) + P(A_1, A_2, \mu) * \frac{dt}{4} * (I_{i+1,j}(t) + I_{i-1,j}(t) + I_{i,j+1}(t) + I_{i,j-1}(t))$$

where we took the lattice to have periodic boundaries. The function $P(A_1, A_2, \mu)$ describes the rate at which an infected cell transmits the infection to its nearest neighbours and decreases monotonically with increasing concentration of 25HC. $P(A_1, A_2, \mu)$ is defined as

$$P(A_1, A_2, \mu) = A_1 e^{-A_2 \mu}$$

where µ denotes the concentration.

Using a lattice of 50x50 cells each of diameter 10µm with simulations of length 10 time steps, we infected one cell at the centre of the dish ($I_{25,25}$=1) with all others uninfected ($I_{i\neq 25, j\neq 25}$=0). We optimised the parameters A_1 and A_2 to best fit the plaque diameters observed for the range of 25HC concentrations in Fig. 5E at the end of the time steps. Plaque sizes were determined by mass of cells with $I_{ij} > 0.5$ and parameters optimised to minimise a least squared difference scoring function using the Genetic Algorithm function in the Global

Optimization Toolbox of Matlab (http://www.mathworks.com), yielding parameter pair (A_1, A_2) = (1.6939, 0.1457).

In this parameter pair, A_1 describes the rate of transmission in the absence of 25HC and A_2 describes the response of transmission to 25HC concentration. Our next task was to estimate how much of this transmission is attributable to a regulation of viral growth and how much is attributable to a regulation of viral entry. We considered $P(A_1, A_2, \mu)$ to be a product of two terms V and C

$$P(A_1, A_2, \mu) = V(B_1, B_2, \mu)C(D_1, D_2, \mu)$$

where

$$V(B_1, B_2, \mu) = B_1 e^{-B_2\mu}$$

is the contribution to infectious transmission from the regulation of viral growth by 25HC and

$$C(D_1, D_2, \mu) = D_1 e^{-D_2\mu}$$

is the contribution from regulation of cell permissivity. From the equations above, we can see that $A_1=B_1D_1$ and $A_2=B_2+D_2$ and by calculating the values of B_2 and D_2 we can assess the relative contributions of the two modes of regulation by 25HC.

Fig. 5A (left panel) shows the amount of viral genome present in cells in the presence of three concentrations of 25HC. At 25HC concentrations of 0, 0.5 and 10uM the copy numbers are 6163.00, 5007.33 and 4483.67, respectively. Taking these values to be proportional to viral entry, we have that

$$\frac{C(D_1, D_2, 0.5uM)}{C(D_1, D_2, 0uM)} = e^{-D_2(0.5)} = \frac{5007.33}{6163.00} \text{ and } \frac{C(D_1, D_2, 10uM)}{C(D_1, D_2, 0uM)} = e^{-D_2(10)} = \frac{4483.67}{6163.00}$$

Optimising D_2 to these expressions with equal weighting, using a least squared difference scoring function and the Genetic Algorithm function in the Global Optimization Toolbox of Matlab, we obtained the value D_2=0.0334. From the equation $A_2=B_2+D_2$, we can now determine that B_2=0.1123. This allows us to write the transmission rate in terms of these two contributions

$$P(A_1, A_2, \mu) = A_1 e^{-B_2\mu - D_2\mu}$$

and we can see from the values of B_2 and D_2 that 25HC regulates both viral growth and cell proliferation. However, because B_2 is 3-4 times greater than D_2, the regulation of viral growth by 25HC is the dominant factor in suppression of viral proliferation by 25HC.

To obtain the simulations of plaque growth shown in Figure S6, we recast the model as a Monte Carlo system in which cells were seeded randomly with a probability of 0.001. This corresponds to an MOI at infection of 0.001. Cell infection levels, I_{ij}, were constrained to take only the values 0 and 1, at each time step, cells were given the opportunity to switch from an uninfected state to an infected state with a probability

$$P(A1, A2, \mu) * \frac{dt}{4} * (I_{i+1,j}(t) + I_{i-1,j}(t) + I_{i,j+1}(t) + I_{i,j-1}(t))$$

Supplemental References

Aerts, S., Van Loo, P., Thijs, G., Mayer, H., de Martin, R., Moreau, Y., and De Moor, B. (2005). TOUCAN 2: the all-inclusive open source workbench for regulatory sequence analysis. Nucleic Acids Res *33*, W393-396.

Angulo, A., Ghazal, P., and Messerle, M. (2000). The major immediate-early gene ie3 of mouse cytomegalovirus is essential for viral growth. J Virol *74*, 11129-11136.

Arthur, J.L., Scarpini, C.G., Connor, V., Lachmann, R.H., Tolkovsky, A.M., and Efstathiou, S. (2001). Herpes simplex virus type 1 promoter activity during latency establishment, maintenance, and reactivation in primary dorsal root neurons in vitro. J Virol *75*, 3885-3895.

Borst, E.M., Benkartek, C., and Messerle, M. (2007). Use of bacterial artificial chromosomes in generating targeted mutations in human and mouse cytomegaloviruses. Curr Protoc Immunol *Chapter 10*, Unit 10 32.

Conesa, A., Nueda, M.J., Ferrer, A., and Talon, M. (2006). maSigPro: a method to identify significantly differential expression profiles in time-course microarray experiments. Bioinformatics (Oxford, England) *22*, 1096-1102.

de Oliveira, A.P., Glauser, D.L., Laimbacher, A.S., Strasser, R., Schraner, E.M., Wild, P., Ziegler, U., Breakefield, X.O., Ackermann, M., and Fraefel, C. (2008). Live visualization of herpes simplex virus type 1 compartment dynamics. J Virol *82*, 4974-4990.

Dolken, L., Ruzsics, Z., Radle, B., Friedel, C.C., Zimmer, R., Mages, J., Hoffmann, R., Dickinson, P., Forster, T., Ghazal, P., and Koszinowski, U.H. (2008). High-resolution gene expression profiling for simultaneous kinetic parameter analysis of RNA synthesis and decay. Rna *14*, 1959-1972.

Dutia, B.M., Roy, D.J., Ebrahimi, B., Gangadharan, B., Efstathiou, S., Stewart, J.P., and Nash, A.A. (2004). Identification of a region of the virus genome involved in murine gammaherpesvirus 68-induced splenic pathology. J Gen Virol *85*, 1393-1400.

Fallaux, F.J., Kranenburg, O., Cramer, S.J., Houweling, A., Van Ormondt, H., Hoeben, R.C., and Van Der Eb, A.J. (1996). Characterization of 911: a new helper cell line for the titration and propagation of early region 1-deleted adenoviral vectors. Hum Gene Ther *7*, 215-222.

Gierasch, W.W., Zimmerman, D.L., Ward, S.L., Vanheyningen, T.K., Romine, J.D., and Leib, D.A. (2006). Construction and characterization of bacterial artificial chromosomes containing HSV-1 strains 17 and KOS. J Virol Methods *135*, 197-206.

Ho Sui, S.J., Fulton, D.L., Arenillas, D.J., Kwon, A.T., and Wasserman, W.W. (2007). oPOSSUM: integrated tools for analysis of regulatory motif over-representation. Nucleic Acids Res *35*, W245-252.

Huang da, W., Sherman, B.T., Zheng, X., Yang, J., Imamichi, T., Stephens, R., and Lempicki, R.A. (2009). Extracting biological meaning from large gene lists with DAVID. Curr Protoc Bioinformatics *Chapter 13*, Unit 13 11.

Irizarry, R.A., Bolstad, B.M., Collin, F., Cope, L.M., Hobbs, B., and Speed, T.P. (2003). Summaries of Affymetrix GeneChip probe level data. NAR *31*, e15.

Kropp, K.A., Robertson, K.A., Sing, G., Rodriguez-Martin, S., Blanc, M., Lacaze, P., Hassim, M.F., Khondoker, M.R., Busche, A., Dickinson, P., *et al.* (2011). Reversible inhibition of murine cytomegalovirus replication by gamma interferon (IFN-gamma) in primary macrophages involves a primed type I IFN-signaling subnetwork for full establishment of an immediate-early antiviral state. J Virol *85*, 10286-10299.

Kropp, K.A., Simon, C.O., Fink, A., Renzaho, A., Kuhnapfel, B., Podlech, J., Reddehase, M.J., and Grzimek, N.K. (2009). Synergism between the components of the bipartite major immediate-early transcriptional enhancer of murine cytomegalovirus does not accelerate

virus replication in cell culture and host tissues. The Journal of general virology *90*, 2395-2401.

Ogundare, M., Theofilopoulos, S., Lockhart, A., Hall, L.J., Arenas, E., Sjovall, J., Brenton, A.G., Wang, Y., and Griffiths, W.J. (2010). Cerebrospinal fluid steroidomics: are bioactive bile acids present in brain? J Biol Chem *285*, 4666-4679.

Ruzsics, Z., Wagner, M., Osterlehner, A., Cook, J., Koszinowski, U., and Burgert, H.G. (2006). Transposon-assisted cloning and traceless mutagenesis of adenoviruses: Development of a novel vector based on species D. J.virol *80*, 8100-8113.

Tischer, B.K., Smith, G.A., and Osterrieder, N. En passant mutagenesis: a two step markerless red recombination system. Methods Mol Biol *634*, 421-430.

Westover, E.J., and Covey, D.F. (2006). Synthesis of ent-25-hydroxycholesterol. Steroids *71*, 484-488.

Zerboni, L., Sommer, M., Ware, C.F., and Arvin, A.M. (2000). Varicella-zoster virus infection of a human CD4-positive T-cell line. Virology *270*, 278-285.

Zhijin Wu, Rafael A. Irizarry, Robert Gentleman, Martinez-Murillo, F., and Spencer, F. (2004). A Model-Based Background Adjustment for Oligonucleotide Expression Arrays. Journal of the American Statistical Association *99*, 909-917.
