# Supplementary figures and images for: The Transcription Factor STAT-1 Couples Macrophage Synthesis of 25-Hydroxycholesterol to the Interferon Antiviral Response
Source: Immunity. 2013 Jan 24;38(1):106–18. doi: 10.1016/j.immuni.2012.11.004 (PMC3556782; doi:10.1016/j.immuni.2012.11.004)

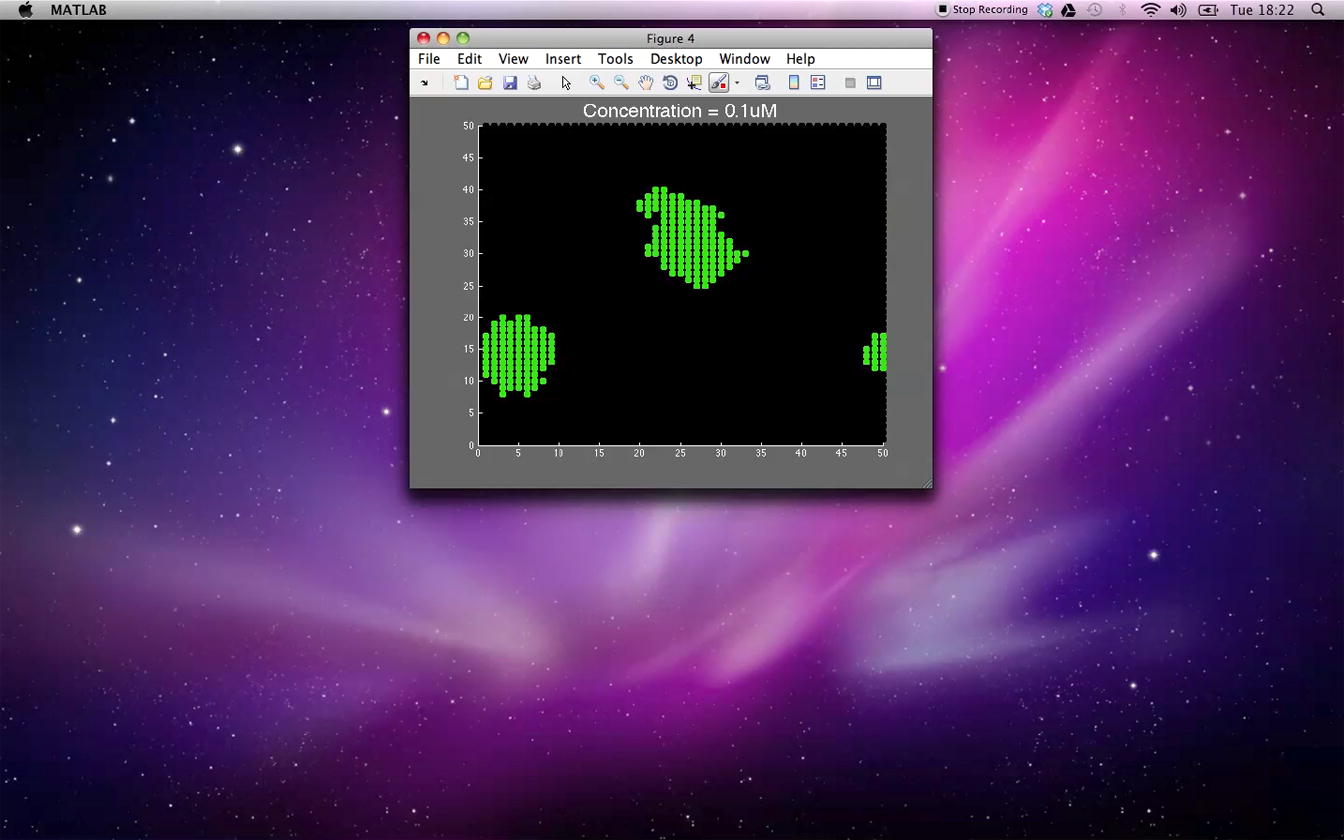

Supplement: Movie S1. Visual Representation of Computational Modeling of Plaque Growth, Related to Figure 5 [file mmc2.jpg]
